# Identification of a new gene signature for prognostic evaluation in cervical cancer: based on cuproptosis-associated angiogenesis and multi-omics analysis

**DOI:** 10.1186/s12935-023-03189-x

**Published:** 2024-01-10

**Authors:** Jiawen Kang, Jingwen Jiang, Xiaoqing Xiang, Yong Zhang, Jie Tang, Lesai Li

**Affiliations:** 1 Department of Gynecologic Oncology, School of Medicine, Hunan Cancer Hospital/The Affiliated Cancer Hospital of Xiangya, Central South University, Changsha, Hunan China; 2 Department of Clinical Medicine, Medical College of Hunan Normal University, Changsha, Hunan China

**Keywords:** Cervical cancer, Cuproptosis, Angiogenesis, Prognosis, Immune infiltration, Cell communication

## Abstract

Patients with recurrent or metastatic cervical cancer are in urgent need of novel prognosis assessment or treatment approaches. In this study, a novel prognostic gene signature was discovered by utilizing cuproptosis-related angiogenesis (CuRA) gene scores obtained through weighted gene co-expression network analysis (WGCNA) of The Cancer Genome Atlas (TCGA) and Gene Expression Omnibus (GEO) datasets. To enhance its reliability, the gene signature was refined by integrating supplementary clinical variables and subjected to cross-validation. Meanwhile, the activation of the VEGF pathway was inferred from an analysis of cell-to-cell communication, based on the expression of ligands and receptors in cell transcriptomic datasets. High-CuRA patients had less infiltration of CD8 + T cells and reduced expression of most of immune checkpoint genes, which indicated greater difficulty in immunotherapy. Lower IC50 values of imatinib, pazopanib, and sorafenib in the high-CuRA group revealed the potential value of these drugs. Finally, we verified an independent prognostic gene *SFT2D1 *was highly expressed in cervical cancer and positively correlated with the microvascular density. Knockdown of *SFT2D1 *significantly inhibited ability of the proliferation, migration, and invasive in cervical cancer cells. CuRA gene signature provided valuable insights into the prediction of prognosis and immune microenvironment of cervical cancer, which could help develop new strategies for individualized precision therapy for cervical cancer patients.

**Supplementary Information:**

The online version contains supplementary material available at 10.1186/s12935-023-03189-x.

## Introduction

Cervical cancer ranks first in the female reproductive tract [1] and consists mainly of squamous and adenocarcinoma types of the cervix. Patients diagnosed with early or locally advanced cervical cancer have achieved some remission and have experienced high survival rates with radical resection or concurrent radiotherapy [2, 3], which have significantly reduced mortality, especially in developing countries and poor regions [4]. Nevertheless, the prognosis and treatment outcomes for patients with refractory cervical cancer, encompassing those afflicted with recurrent, persistent, or metastatic forms, remain disheartening [5–7]. The implementation of novel scientific methodologies and an enhanced comprehension of tumor pathogenesis are poised to augment our understanding of cervical cancer mechanisms and ultimately ameliorate the prognosis for individuals grappling with refractory cervical cancer. It is necessary to explore new ways to improve the prognosis of patients with refractory cervical cancer.

Tumor angiogenesis, inflammatory infiltration of the tumor microenvironment, and programmed cell death processes have been identified as contributing factors to tumor metastasis [8]. Tumor cells exhibit elevated secretion of pro-angiogenic factors, which stimulate the development of heterogeneous and immature neovascularization. This heteromorphic neovascularization often leads to a hypoxic microenvironment caused by inadequate perfusion, thereby favoring the survival and growth of more aggressive tumor cells [9]. Simultaneously, the presence of pro-angiogenic factors within the tumor microenvironment facilitates the process of angiogenesis and immunosuppression [10]. Consequently, angiogenesis fosters the tumor’s ability to evade the immune system and engenders drug resistance. Tumor angiogenesis stands as a contributing factor to recurrence, prompting the clinical utilization of anti-angiogenic drugs in the management of advanced or recurrent cervical cancer, resulting in notable enhancements in survival rates [11]. However, the limited applicability of targeting mature stable vessels [12] and the presence of various treatment-related side effects have necessitated the exploration of novel therapeutic approaches [13]. Notably, the remarkable effectiveness of immunotherapy in cervical cancer has highlighted the significance of targeting angiogenesis in the tumor microenvironment for immunotherapeutic interventions [14, 15]. Consequently, for patients with metastatic, persistent, and recurrent cervical cancer who exhibit PD-L1 positivity, the combination of pablizumab and chemotherapy, with or without bevacizumab, has emerged as the preferred first-line treatment option [6]. Investigating modifications in the immune microenvironment and immune checkpoint genes within tumors experiencing varying angiogenic states can provide valuable insights into the development of precise combinations of vascular targeting therapy and immunotherapy.

Recent findings indicate that several well-established regulators of programmed cell death play a role in promoting angiogenesis [8, 16–20]. Additionally, cuproptosis, a distinct form of programmed cell death, is primarily characterized by the excessive accumulation of intracellular copper, leading to cell death [21]. It has been shown increased intratumor copper concentrations promote tumor growth and invasion as well as treatment resistance [22]. Serum copper concentrations have been found to exhibit a correlation with tumor progression and morbidity [23]. Additionally, cuproptosis, a recently identified mode of cell death, has been reported to play a role in tumor growth, angiogenesis, and tumor metastasis [24, 25]. Studies have demonstrated that copper facilitates tumor angiogenesis by activating various angiogenic factors, such as basic fibroblast growth factor (bFGF) and vascular endothelial growth factor (VEGF) [26]. Moreover, copper is implicated in signal transduction processes within endothelial cells, thereby influencing angiogenesis [23]. In summary, copper assumes an indispensable function in the advancement of tumors as a trace element crucial for the proliferation of cancer cells and the formation of blood vessels within tumors. Further exploration of the correlation between angiogenesis and cuproptosis is imperative, as it holds potential for novel treatment approaches [19, 27]. The association between angiogenesis and cuproptosis in cervical cancer has yet to be investigated, thus necessitating a meticulous and proactive investigation employing innovative methodologies.

The emergence of precision oncology and the integration of big data have facilitated the utilization of bulk RNA sequencing to uncover the mean gene expression in tissues, thereby enabling exploration into the realm of cognitive differential gene expression [28]. Furthermore, the progression of technological tools has allowed for the implementation of single cell sequencing, which has proven instrumental in discerning differential gene expression among cells and investigating intricate cell populations [29–31]. This technique has significantly contributed to the fields of tumor diagnosis, targeted therapy, and prognosis prediction [32, 33]. The analysis of intercellular communication in cell populations aids in the elucidation of communication and signaling mechanisms among diverse cells [34]. Additionally, correlation analysis of receptor-ligand pairs enables a deeper comprehension of cellular functionality and regulatory networks. In this study, we have developed a prognostic model for the CuRA gene using both single-cell RNA sequencing and bulk RNA sequencing. This model holds promise for the development of innovative prognostic prediction models and treatment approaches for individuals with cervical cancer.

## Materials and methods

### Data acquisition

The cervical cancer patient dataset (the TCGA-CESC cohort) was obtained from the Cancer Genome Atlas as the training group (TCGA, https://portal.gdc.cancer.gov/). For external validation, we used 55 cervical cancer patients from the GSE52903 dataset in Gene Expression Omnibus (GEO, https://www.ncbi.nlm.nih.gov/geo/) databases. We also included single-cell sequencing datasets GSE168652 in cervical cancer using “Seurat” package [35] for calculating genetic correlations to score cells and patients. The scores of CuRA gene-sets were calculated by applying the “Percentage FeatureSet” function. The GeneCards website (https://www.genecards.org/) was used to obtain 1245 angiogenesis-related genes using the “angiogenesis” keyword with a correlation > 1. R package “limma” [36] was used to obtain cuproptosis-related angiogenesis (CuRA) genes. Genes with differential expression in normal and cervical tissues were obtained from the GEPIA website (http://gepia.cancer-pku.cn/).

### WGCNA

Single-sample gene set enrichment analysis (ssGSEA), which estimated the relative enrichment of a particular gene set in each sample by comparing the gene expression data of that sample with that set. We performed weighted gene co-expression network analysis (WGCNA) using the “WGCNA” package [37]. The threshold of clustering cut tree was set to 210, and the minimum threshold was set to 80. We merged the modules with threshold < 0.5. Then, we performed analysis and included all genes in modules of CuRA phenotypes with *P* < 0.05 for subsequent analysis.

### Construction of the CuRA model

We configured the alpha parameter of the elastic network to 0.5 and computed the errors for ridge regression, lasso regression and elastic network regression. The model regression was constructed using the “glmnet” package [38]. The “timeROC” package [39] and “survivalROC” package [40] were performed to plot ROC curves for survival outcomes at different time points. Nomogram based on logistic regression and Cox regression was constructed using the “rms” package [41].

### Immune infiltration analysis

“IOBR” package [42] was used to immune infiltration analysis. Six algorithm CIBERSORT, EPIC, MCP, XCELL, TIMER, QUANTISEQ was performed to compare the differences between the high and low-CuRA groups. Differences in the expression of immune checkpoint genes were also compared. The relevant mutation data were obtained from Cbioportal (https://www.cbioportal.org/datasets). The “maftools” package [43] was performed for visualization.

### Cell communication analysis

We performed cell communication analysis using the R package “CellChat” [34]. We filtered out cell communication with less than 10 cells and obtained the cell communication relationship between each cell. We inferred cell-to-cell communication at the pathway level, deduced pathway-level interaction networks, and obtained the interaction relationship between receptor-ligand pairs and cell communication.

### GSEA

The GSEA software (version 3.0) was downloaded from the GSEA (http://software.broadinstitute.org/gsea/index.jsp) website, divided the samples into high and low expression groups based on the expression levels of *SFT2D1*. A *P *value of < 0.05 and an FDR value of < 0.25 were considered statistically significant. The corresponding data was listed in Additional file 1: Table S5.

### Drug sensitivity analysis

We searched the GDSC database to predict drug sensitivity by comparing the IC50 of drugs among different groups based on the CuRA scores. By analyzing in the Drug Signatures Database (DSigDB, http://tanlab.ucdenver.edu/DSigDB), we listed corresponding small molecule drugs of relevant modeling genes (Additional file 1: Table S4).

### Cell culture

The ECT1/E6E7 cell line (ATCC: CRL-2614™), the SiHa cell line (ATCC: HTB-35™), the CaSki cell line (ATCC: CRM-CRL-1550™) were obtained from American Type Culture Collection (ATCC). Ect1/E6E7 cells, SiHa cells were cultured in DMEM medium (Procell, Wuhan,China) containing 10% fetal bovine serum (Procell, Wuhan, China), 100 U/mL penicillin and 100 µg/mL streptomycin, and incubated at 37 °C under conditions of 5% CO_2_. CaSki cells were cultured with the same conditions in 1640 medium (Procell, Wuhan, China). These cells were transfected with synthetic small interfering RNAs (GenePharma, Shanghai, China) by Lipo8000™ Transfection Reagent (Beyotime, Shanghai, China), and the siRNA sequences targeting *SFT2D1 *gene are provided in the Additional file 1: Table S6.

### Real-time fluorescence quantitative PCR

We extracted total RNA of cells using TRIZOL reagent (Vazyme, China), followed by adding chloroform for centrifugation. The supernatant was collected and mixed with isopropanol. The RNA pellet was washed with 75% ethanol and air-dried. The purity of RNA was measured using a spectrophotometer (Thermo Fisher Scientific, USA). The cDNA was synthesized using reverse transcription reagent (TransGen, China) for fluorescence quantification (RT-qPCR).

### Immunohistochemistry

We collected normal cervical tissue and cervical cancer tissue at the Hunan Provincial Cancer Hospital for immunohistochemical staining, and it has been reviewed and approved by the Ethics Committee of Hunan Cancer Hospital. After dewaxing of sections, heat antigen retrieval was performed. The primary antibodies SFT2D1 (Immunoway, USA, 1:100) and CD31 (ZenBio, China, 1:100) were incubated overnight at 4 °C. The secondary antibodies were incubated for 20 min using the PV-9000 kit (ZSGB-BIO, China). DAB reagent (ZSGB-BIO, China) was used for antibody staining, with brown-yellow indicating positive signal areas. Cell nuclei were stained blue with hematoxylin (Servicebio, China). Images were captured using microscope (Zeiss, Germany) and analyzed using Image J software (1.53, USA).

### Western blotting

We added a mixture of cell lysis buffer (Servicebio, China) and protease inhibitor PMSF to the cells. The sample was then denatured by adding SDS-loading buffer and subjected to electrophoresis. The PVDF membrane (Millipore, USA) was wet-transferred at a constant current. After blocking with skim milk at room temperature for 2 h, the primary antibody SFT2D1 (Immunoway, USA, 1:1000) was incubated at 4℃ overnight. The secondary antibody (bioworld, USA, 1:10000) was incubated at room temperature for 1 h, followed by detection with a developing solution.

### CCK-8 assay

After transfection, the appropriate amounts of resuspended cervical cancer cells in the logarithmic phase of growth were added in 96-well plates with trypsin digestion down and set up 5 sub-wells per group (NC, si-*SFT2D1*). When the cells were adhered to the wall, the solution in Cell Counting Kit-8 (CCK-8, APE, USA) was added after replacing the fresh medium, and the absorbance value was measured at 450 nm after incubation with the cells for 2 h at 37 ℃ in an MicroplateReader Instrument (Biotek, USA). First data were grouped into the 0 h group. And the readings of 0 h, 24 h, 48 h, 72 and 96 h were recorded to calculate the proliferative capacity of the cells.

### Wound scratch experiment

After transfection, resuspended cells were added in 6-well plates by trypsin digestion. The cells incubated under conditions of constant temperature and constant CO_2_, a straight line was drawn vertically in the center of the 6-well plate with the pipette tip, and then the width of the straight line was photographed and recorded under the microscope. Replace the medium with serum-free medium to continue incubation for 24-48 h, and then take pictures with the microscope to record the growth of cells. Cell migration rate = (0 h scratch width - scratch width after incubation)/0 h scratch width × 100%, which was analyzed by ImageJ software (1.53, USA) to calculate the migration ability of cells.

### Transwell migration and invasion assay

The chambers were hydrated with serum-free DMEM medium for 30 min. After aspirating the medium a cell suspension mixed with appropriate amount of serum-free medium was added to the upper chamber, and the lower chamber was incubated with medium containing 10% serum for 24 h. After fixation in methanol and staining with crystal violet, the cells that did not pass through the upper chamber were wiped away, and the cells that passed through the lower chamber were observed and counted under the microscope, and the migration ability of the cells was judged according to the number of cells. The cells were observed and counted in the lower chamber under the microscope. The matrix gel was purchased from Corning (USA). The gel was spread on the upper chamber surface, and after 2 h, the gel was allowed to solidify and then hydrated with medium, and the same procedure was followed to determine the invasion ability of the cells according to the number of cells.

### Statistical analyses

We used R software (version 4.2) and GraphPad prism (version 8.3.0) for relative analyses and drawings. T-test was performed to analysis differences between two groups. ANOVA was used to analysis differences between three or more groups. *P* < 0.05 was considered as statistically different.

## Results

### Flow chart

**Fig. 1:**
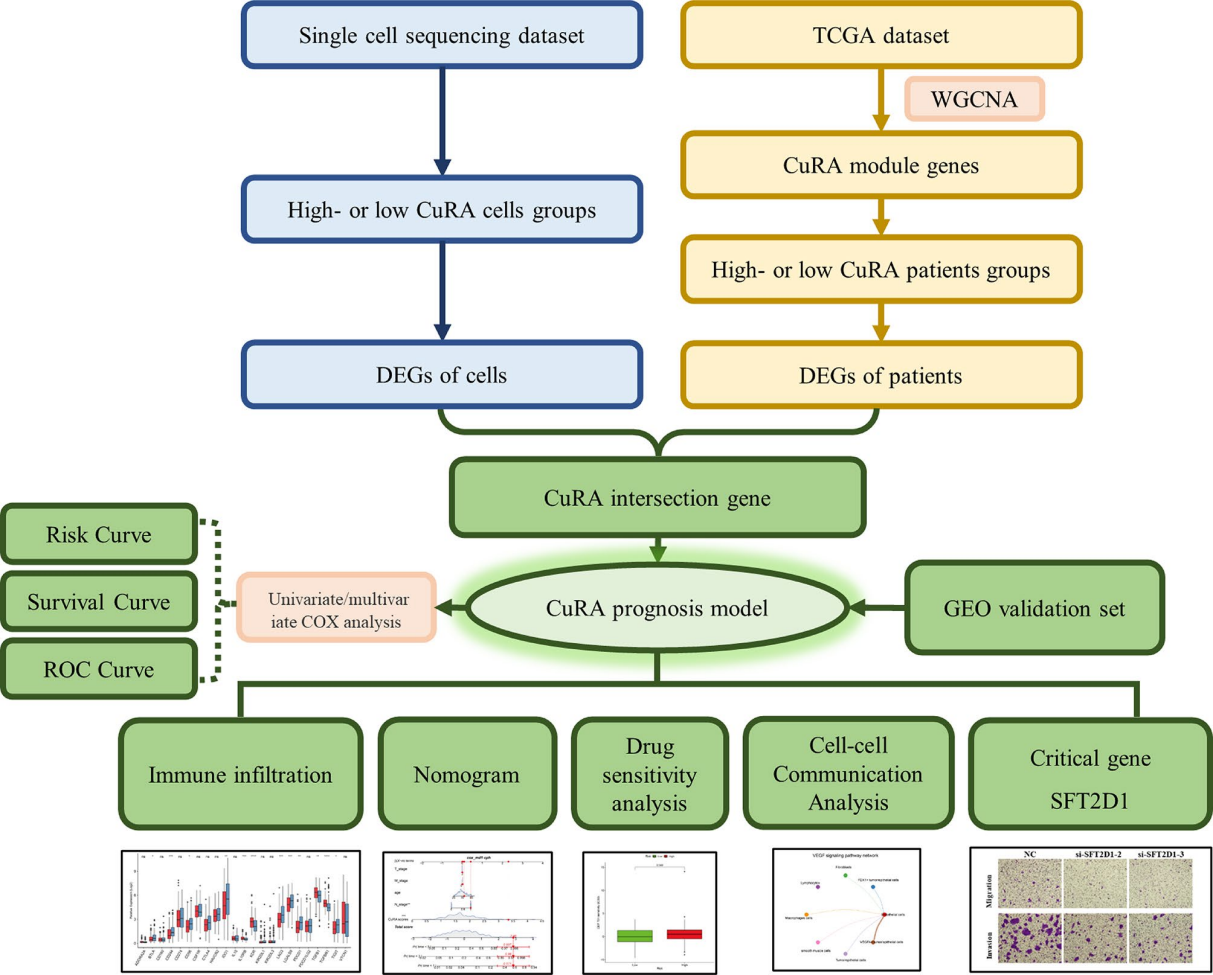
Flow chart of the full text. CuRA, cuproptosis-related angiogenesis gene. DEGs, differential genes. WGCNA, weighted correlation network analysis. ROC, receiver operating characteristic

The flow chart was shown in Fig. 1.

### Identification of phenotype -related different CuRA genes by WGCNA

**Fig. 2:**
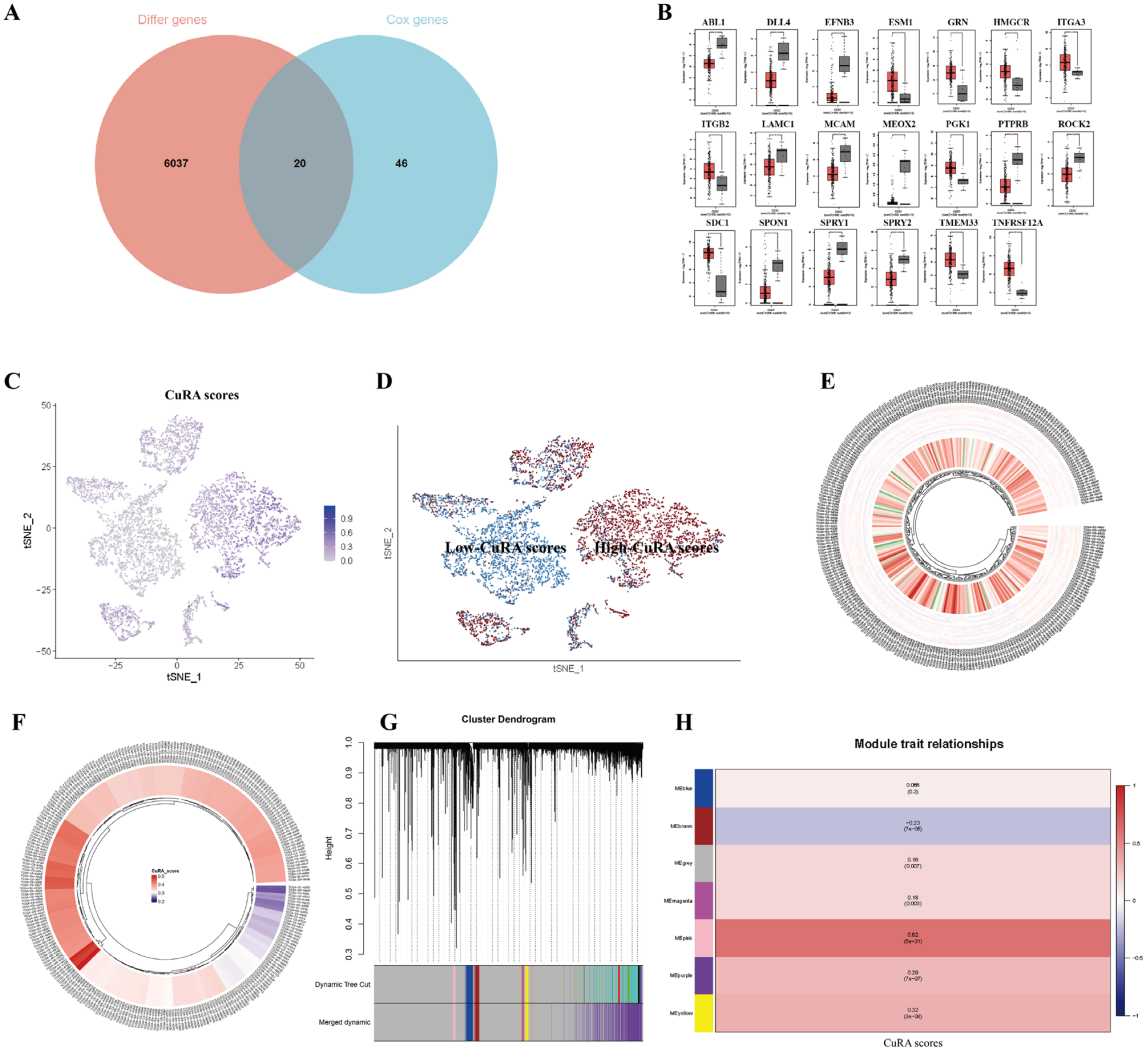
Selection of CuRA phenotype-related genes by WGCNA. (**A**) Venn diagram of intersection of 6057 differential genes with 66 prognostic genes. (**B**) Histogram of 20 CuRA genes validated at the GEPIA website. (**C**) Scores of CuRA genes in GSE168652, with the darker the purple, the higher the scores. (**D**) Grouping into high-CuRA and low-CuRA groups, red indicating high-CuRA group, blue indicating low-CuRA group. (**E**) Quantification of immune infiltration levels by ssGSEA. (**F**) Circle plot of CuRA genes scores of patients. (**G**) Waterfall plot of WGCNA. (**H**) Heatmap of phenotype-related modules

19 cuproptosis genes was shown in Additional file 1: Table S1. Then we downloaded angiogenesis-associated genes with correlation > 1 from the GENECARD website. Based on the gene expression of TCGA-CESC patients, as a screening condition of |cor|>0.3 and *P* < 0.05, finally 533 CuRA genes were included. At the same time, we performed univariate cox analysis to obtain 66 prognostic CuRA genes (Additional file 1: Figure S1) from 533 genes. To explore genes that are differentially expressed between the normal cervix and cervical cancer, we downloaded 6057 differential genes from the GEPIA website(|log_2_FC|>1). We took the intersection of 6057 differential genes with the 66 prognostic CuRA genes (Fig. 2A) above and finally obtained 20 CuRA genes with significant differences (Fig. 2B, Additional file 1: Table S2). Based on the scores of 20 CuRA gene-sets, each cell was divided into high-CuRA and low-CuRA cells groups according to the median value in GSE168652 dataset (Fig. 2C, D). In the TCGA-CESC cohort, we quantified and visualized the level of immune infiltration in different patients by ssGSEA (Fig. 2E). Meanwhile, we performed CuRA scores for each patient by the ssGSEA algorithm, then plotted circle plots to see the differences in scoring for each patient (Fig. 2F). WGCNA was performed to obtain phenotype-related modules for CuRA genes (Fig. 2G). We included all genes (*P* < 0.05) in non-grey modules (The grey module contained genes that couldn’t be classified as any module): pink, brown, magenta, purple, and yellow modules for the follow-up study (Fig. 2H).

### Construction of a CuRA prognostic model

**Fig. 3:**
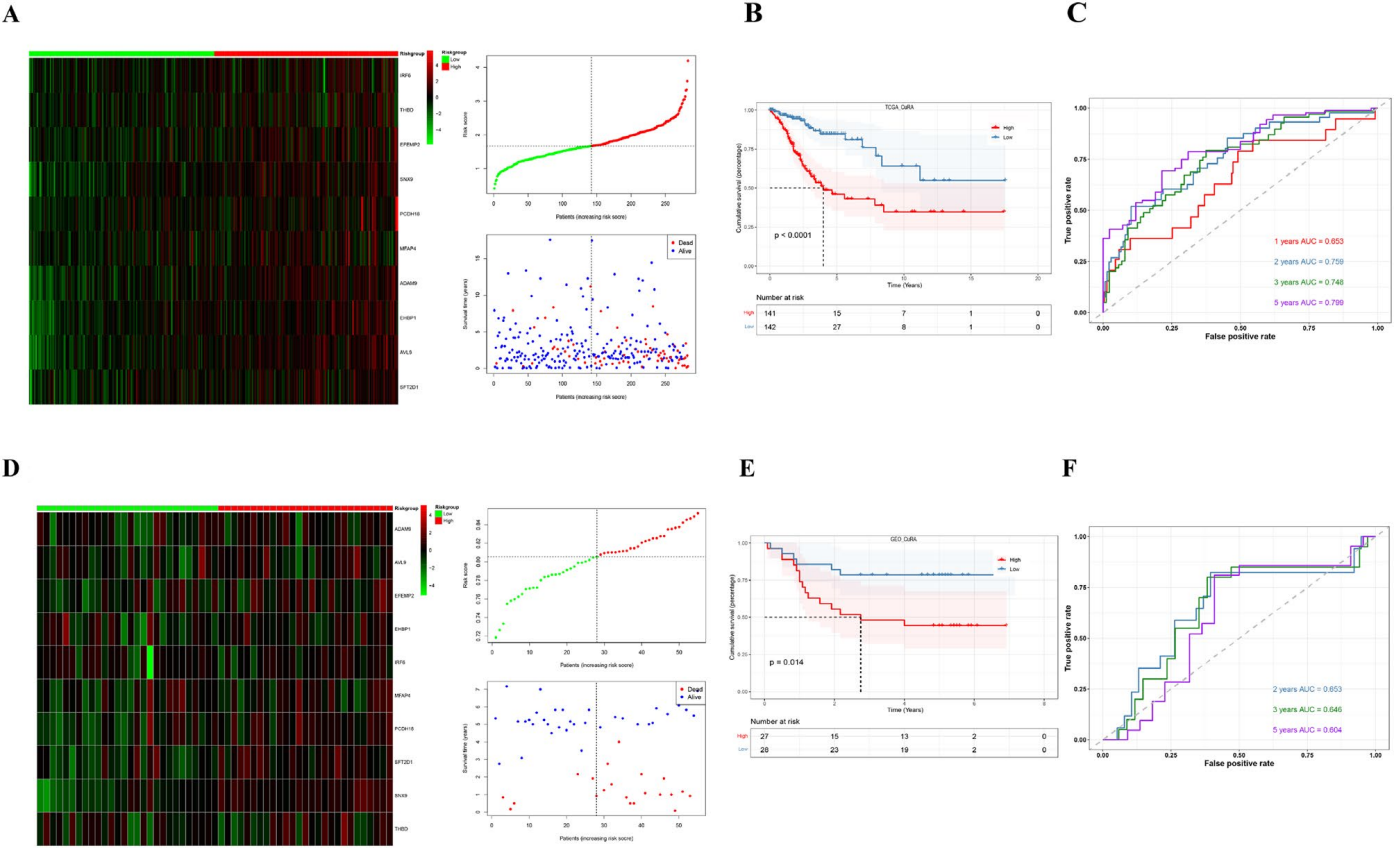
Construction and verification of a CuRA prognostic model. Heatmap and point chart of TCGA patients (**A**) and GEO patients (**D**). Survival curve plot of TCGA patients (**B**) and GEO patients (**E**). ROC curve of TCGA patients (**C**) and GEO patients (**F**)

We obtained CuRA intersection genes by intersecting the different genes of high-CuRA and low-CuRA cells groups of GSE168652 with the WGCNA phenotype-related modular genes of patients. We configured the alpha parameter of the elastic network to 0.5 and computed the errors for primary methods. The results revealed that the error for ridge regression is 3.055991, for lasso regression it is 0.0002452, and for elastic network it is 0.0002547035. After comparing the errors of the regression methods, we opted for lasso regression to construct the model (Additional file 1: Figure S2). Then a prognostic model was constructed based on 10 CuRA genes by lasso regression according to optimal lambda value (Additional file 1: Figure S3A, B). CuRA modeling scores = 0.001007113*IRF6+ 0.001993273*THBD+ 0.007235823*EFEMP2+ 4.45E-04*SNX9+ 0.012465781*PCDH18+ 7.23E-05*MFAP4+ 0.005812479*ADAM9+ 0.004983918*EHBP1+ 0.055328306*AVL9+ 0.00770984*SFT2D1. We included the GSE52903 as validation set. Then we analyzed the differences between TCGA patients (Additional file 1: Figure S3C) and GSE52903 patients (Additional file 1: Figure S3D) according to CuRA modeling scores by PCA. Further, we assessed efficacy of the model to predict prognosis. Heatmaps and point chart of risk scores were drawn showing differences in expression of CuRA model genes in TCGA patients (Fig. 3A) and GEO patients (Fig. 3D. In TCGA dataset (Fig. 3B) and GEO dataset (Fig. 3E), patients in the high-CuRA group had significantly lower survival than those in the low-CuRA group. In the ROC curves, the 1, 2, 3, and 5-year AUC values were 0.653, 0.759, 0.748, and 0.799 for TCGA patients, respectively (Fig. 3C). The AUC values for 2, 3, and 5-year survival for GEO patients were 0.653, 0.646, and 0.604, respectively (Fig. 3F). Results indicated the model had better predictive effect on prognosis in both TCGA and GEO datasets. Meanwhile, we included clinical data of TCGA patients and then combined T1-2 patients into early stage and T3-4 stage patients into late stage of T-stage for univariate COX analysis, and we found CuRA modeling scores, N_stage were risk factors (Additional file 1: Figure S3E). Further, we performed a multivariate COX analysis and the results showed CuRA modeling scores, N_stage, and T_stage were independent risk factors (Additional file 1: Figure S3F).

### Immune infiltration landscape and mutational landscape

**Fig. 4:**
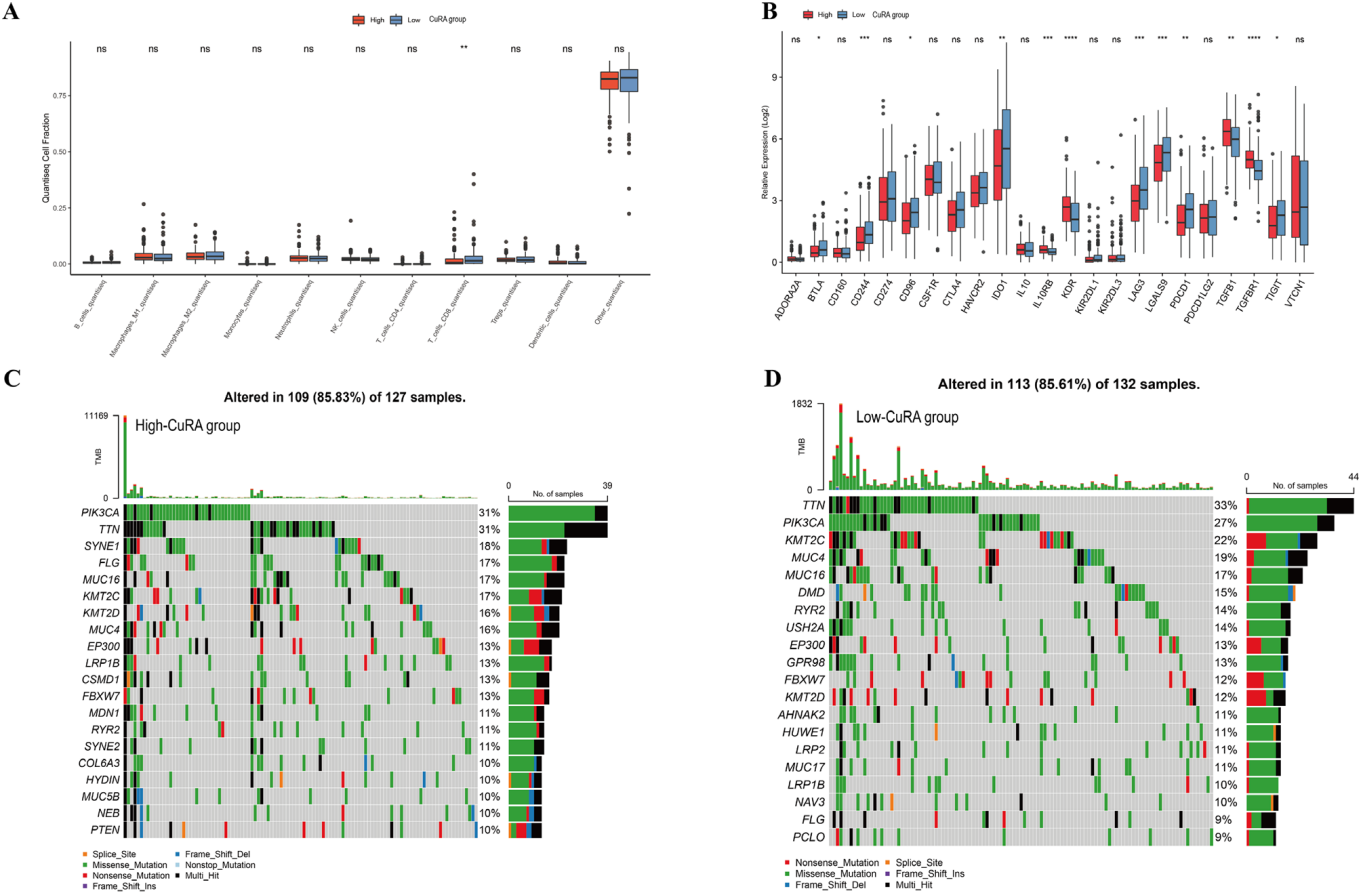
Immune infiltration and tumor mutation analysis. (**A**) Differences in infiltration of 10 types of immune cells between two CuRA groups. (**B**) Differences in expression of immune checkpoints gene between the two groups. (**C**) Tumor mutation characteristics in the high-CuRA group. (**D**) Tumor mutation characteristics in the low-CuRA group

Based on the modeling gene scores, we explored the differences in immune infiltration and tumor mutations of high-CuRA and low-CuRA groups. We introduced six algorithms to perform a comprehensive analysis of immune cell infiltration in two groups (Additional file 1: Figure S5). The six algorithms showed infiltration of the overall immune cells was significantly lower in the high-CuRA group than in the low-CuRA group. Among them, it showed a significant difference between the two groups by xCell algorithm. Next, we compared the 10 types of major immune cells, and found CD8 + T cells were significantly reduced in patients in the high-CuRA group (Fig. 4A). Also, we analyzed the expression of common immune checkpoint genes, and we found the clinically common immune checkpoint genes *CD274*(*PD-L1*) and *CTLA4 *did not differ significantly between two groups (Fig. 4B). Therefore, we speculate patients classified into high-CuRA and low-CuRA groups may not differ in treatment benefit by applying *PD-L1 *inhibitors or *CTLA4 *inhibitors. Also, the results showed most of the immune checkpoint genes were significantly less expressed in the high-CuRA group than in the low-CuRA group. We also found *IL10RB*, *KDR*, *TGFB1*, and *TGFBR1 *genes were significantly more expressed in the high-CuRA group than in the low-CuRA group. Results suggested patients in the high-CuRA group may get better therapeutic outcomes by using inhibitors targeting these 4 genes. Next, we analyzed the mutation landscape in both groups (Fig. 4C, D), and found the top 3 genes with the highest mutation frequencies were *PIK3CA*(31%), *TTN*(31%), *SYNE1*(18%) in 127 patients of the high-CuRA group, while in the low-CuRA group, the top 3 genes with the highest mutation frequencies were *TTN*(33%), *PIK3CA*(27%), *KMT2C*(22%) in 113 patients. We found *SYNE1 *showed a higher mutation frequency (23 cases) in the high-CuRA group and a lower mutation frequency (11 cases) in the low-CuRA group with significant difference between the two groups (odds ratio (OR) = 0.412) (Additional file 1: Figure S6). Also, other genes such as *RELN*, *SPATA31D1*, *TCOF1 *had higher mutations in the high-CuRA group.

### Analysis of clinical characterization and construction of nomogram

**Fig. 5:**
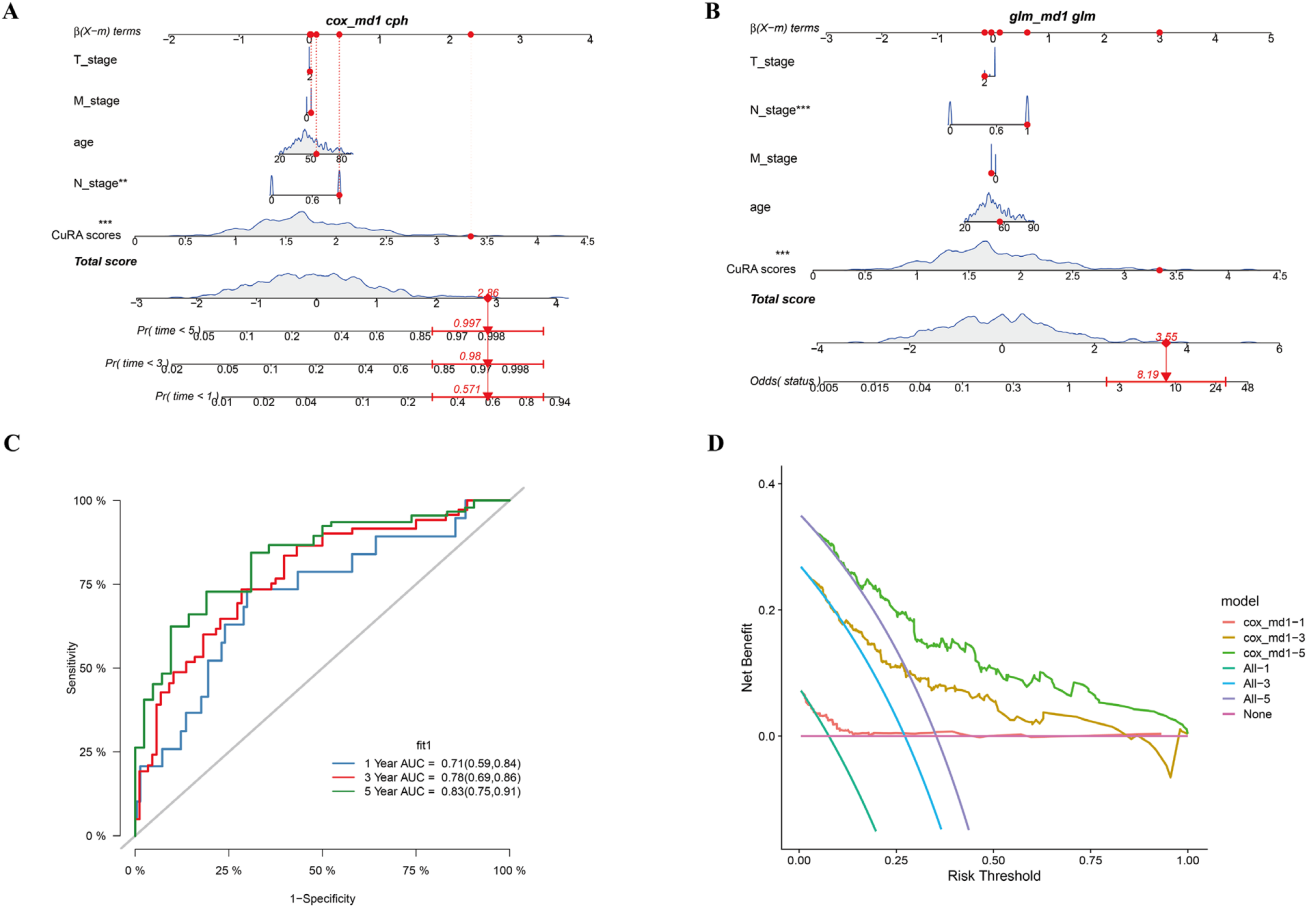
Construction and validation of nomogram. (**A**) Cox regression to construct nomogram. (**B**) Logistic regression to construct nomogram. (**C**) Curves of ROC for 1, 3, and 5-years. (**D**) DCA decision curves for 1, 3, and 5-years

We analyzed prognosis with different clinical characteristics, and constructed a nomogram based on the CuRA model. Results showed the N_stage and CuRA modeling scores contributed significantly to the model. The predicted mortality of the patient was 0.571, 0.98, and 0.997 at 1, 3, and 5-years, respectively (Fig. 5A), and its odds ratio of status was 8.19 (Fig. 5B). ROC curves were performed to predict the accuracy of nomogram. AUC values at 1, 3, and 5-years were 0.71, 0.78, and 0.83, respectively, which indicated the nomogram had good predictive accuracy (Fig. 5C). Finally, we plotted the DCA decision curves, and results showed the model had a strong predictive effect on the survival rate at 1, 3, and 5-years (Fig. 5D).

### Drug sensitivity prediction

**Fig. 6:**
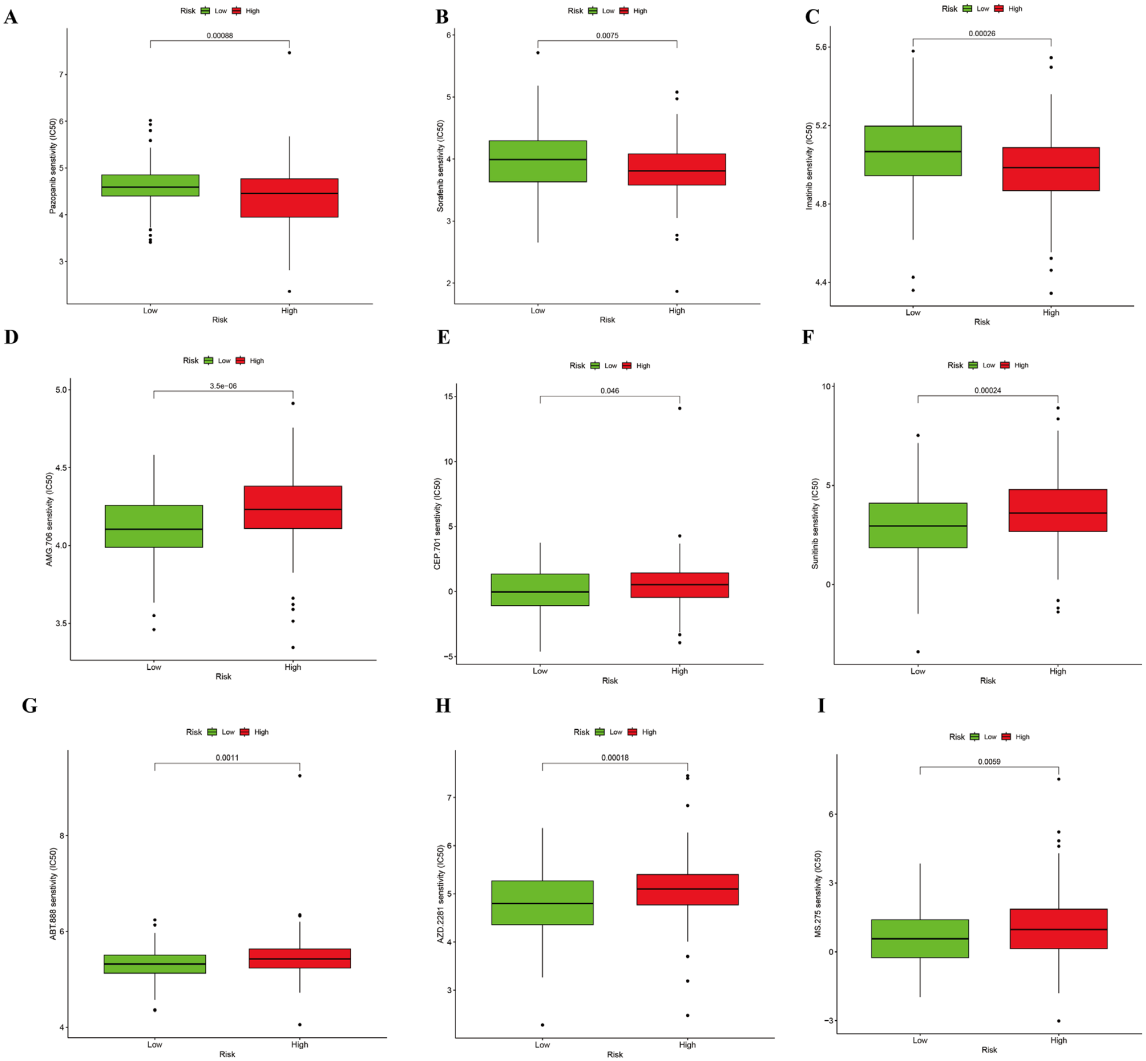
Drug sensitivity analysis of the high and low-CuRA group. (A-I) The IC50 of Imatinib, Pazopanib, and Sorafenib was lower in the high-CuRA group than for the low-CuRA group contrary to AMG.706, CEP.701, Sunitinib, ABT.888, AZD.2281, and MS.275. The vertical coordinates displayed as drug names, demonstration of drugs with statistical significance

To explore the different drugs that may be effective against treating the high- and low-CuRA groups, we used GDSC database by predicting the IC50 to determine the differences in drug sensitivity between the high- and low-CuRA groups. Results showed the IC50 of Imatinib, Pazopanib, and Sorafenib was significantly lower for the high-CuRA group than for the low-CuRA group, suggesting they may have better efficacy when applied with the high-CuRA patient group (Fig. 6A–C). Similarly, for the low-CuRA group, the application of AMG.706, CEP.701, Sunitinib, ABT.888 (Veliparib), AZD.2281 (Olaparib), and MS.275 (Entinostat) may lead to better therapeutic remission (Fig. 6D–I).

### Cell communication analysis

**Fig. 7:**
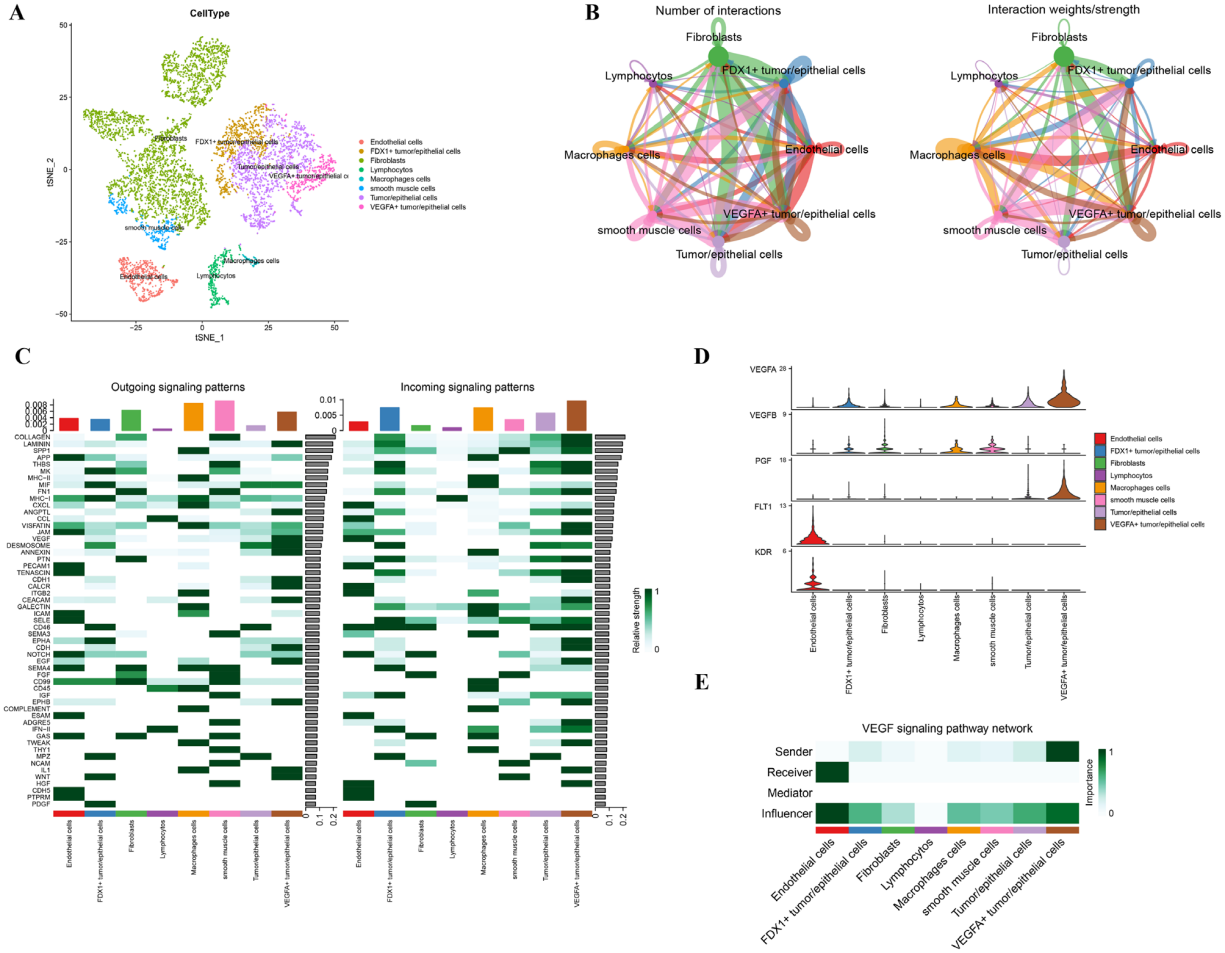
Analysis of cellular communication network. (**A**) Cell annotation of 8 types of cells. (**B**) Number of interactions and interaction weights of samples in GSE168652. (**C**) Schematic diagram of the incoming and outgoing signals of samples. (**D**) Visualization of the expression of key genes in the VEGF signaling pathway in 8 types of cells. (**E**) Visualization of cells involved as senders, receivers, mediators and influencers in the VEGF pathway

We performed cell communication analysis by single-cell sequencing dataset GSE168652 from GEO. We grouped the cells into 25 clusters and divided the cell clusters into 8 types based on annotation, which are: endothelial cells, FDX1 + tumor/epithelial cells, fibroblasts, lymphocytes, macrophages, smooth muscle cells, tumor/epithelial cells (other types), and VEGFA + tumor/epithelial cells (Fig. 7A). We observed the expression and localization of 10 modeling genes in GSE168652 (Additional file 1: Figure S4). Since *FDX1 *was a representative gene for cuproptosis, we labeled FDX1-positive or VEGFA-positive tumor/epithelial cells here in the hope of exploring the relationship between cuproptosis and angiogenesis-associated tumor/epithelial cells in intercellular communication. Multidirectional cell communication was discovered in each cell subpopulation (Fig. 7B). Then we identified the cell-extrinsic communication patterns. We analyzed the signaling pathways of both incoming and outgoing signals in the samples. Our results revealed the main outgoing signals of FDX1 + tumor/epithelial cells were PDGF, WNT, CD46, MHC-1, MIF, and MK pathways, while the primary incoming signals were IFN-II and other pathways. As for VEGFA + tumor/epithelial cells, the major outgoing signals were WNT, EGF, and VEGF, while the main incoming signals involved numerous signaling pathways, including COLLAGEN (Fig. 7C). Specifically, we focused on the involvement of VEGF pathway in cell communication as the key pathway of angiogenesis. We observed higher expression of the key gene VEGFA in the VEGFA + tumors/epithelial cells, tumor/epithelial cells, and FDX1 + tumors/epithelial cells in the VEGF signaling pathway (Fig. 7D). In signal transduction, by calculating the network centrality indices for each cell population, we found VEGFA + tumor/epithelial cells were the dominant signaler in the intercellular communication network, endothelial cells were the main receivers and influencer, and FDX1 + tumors/epithelial cells also played an important role in influencer (Fig. 7E). In the visual circular and hierarchical plots of the VEGF signaling pathway (Additional file 1: Figure S9), our analysis revealed VEGFA + tumor/epithelial cells and FDX1 + tumor/epithelial cells had the most significant effect on endothelial cells. In addition, we analyzed relevant receptor-ligand pairs in FDX1 + or VEGFA + tumors/epithelial cells communicating with other cells respectively (Additional file 1: Figure S10).

### Critical functional role of SFT2D1 in cervical cancer

**Fig. 8:**
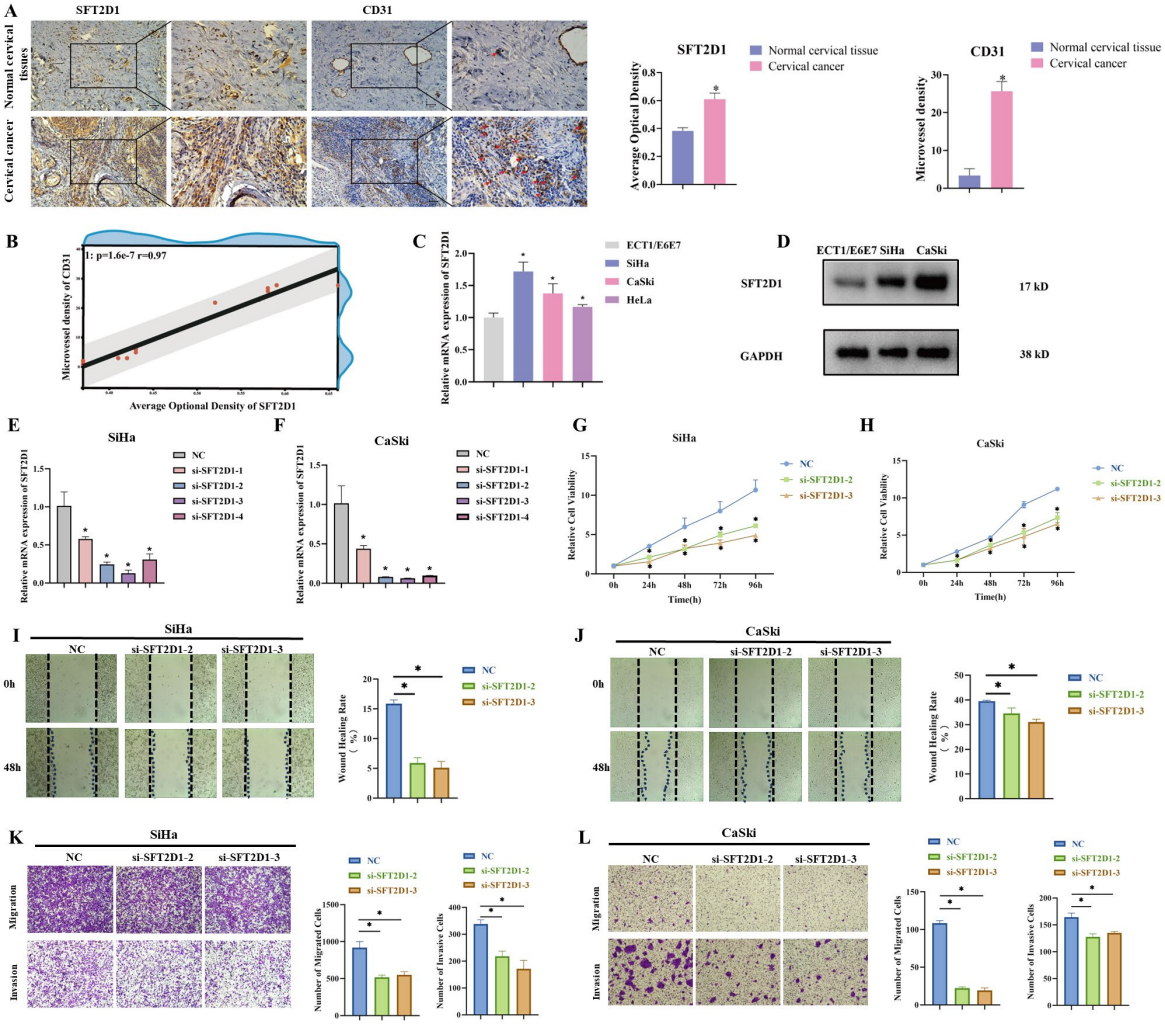
*SFT2D1 *was involved in the progression of cervical cancer. (**A**) SFT2D1 and CD31 were highly expressed in cervical cancer by IHC. (**B**) Correlation analysis between SFT2D1 and CD31. (**C**) Validation of *SFT2D1 *expression by RT-qPCR. (**D**) Validation of SFT2D1 expression by western blotting. Four siRNAs suppressed the expression of *SFT2D1 *in SiHa (**E**)and CaSki (**F**) cells by RT-qPCR. CCK-8 assay in in SiHa (**G**) and CaSki (**H**) cells. Wound scratch assay in SiHa (**I**) and CaSki (**J**) cells. Transwell assay in SiHa (**K**) and CaSki (**L**) cells

We selected prognosis-related model genes among 10 model genes (Additional file 1: Figure S7). *ADAM9*, *EHBP1*, and *SFT2D1 *gene were shown significantly affecting the survival of patients. Meanwhile, we also identified *SFT2D1 *as an independent risk factor by multivariate analysis along with clinical features (Additional file 1: Table S3). Then we performed GSEA of *SFT2D1*, and results showed *SFT2D1 *was mainly involved in the regulation of autophagy, glycosaminoglycan degradation, RNA degradation, riboflavin metabolism, mTOR signaling pathway. We then investigate the effect of *SFT2D1 *on immune microenvironment. The total scores, stromal scores and immune scores were significantly lower in the high-*SFT2D1 *group than in the low-*SFT2D1 *group, and the immune related scores were negatively correlated with *SFT2D1 *expression (Additional file 1: Figure S8). Meanwhile, we performed immunohistochemical analysis of SFT2D1 and the neovascularization marker CD31 in paraffin sections of cervical cancer patients. The results showed both SFT2D1 and CD31 were expressed up-regulated in cervical cancer tissues (Fig. 8A). Correlation analysis also showed positive correlation in SFT2D1 and CD31 (Fig. 8B). Then we performed RT-qPCR to verify *SFT2D1 *was highly expressed in cervical cancer cell lines compared to normal cervical cell lines ECT1/E6E7 (Fig. 8C). Meanwhile, western blotting showed that SFT2D1 was upregulated in SiHa, CaSki cervical cell lines (Fig. 8D). Since the role of *SFT2D1 *in cervical cancer has not yet been explored, we authenticated the effect of *SFT2D1 *on the function of SiHa and CaSki cells by in vitro experiments. RT-qRCR showed that four siRNAs significantly suppressed the expression of *SFT2D1 *in transfected SiHa and CaSki cells (Fig. 8E, F). We selected the two siRNAs with the highest knockdown efficiency among them: si-*SFT2D1*-2, si-*SFT2D1*-3 for subsequent experiments. CCK-8 analysis showed that knockdown of *SFT2D1 *significantly inhibited the proliferative ability of SiHa and CaSki cells (Fig. 8G, H). Wound scratch assay showed that knockdown of *SFT2D1 *significantly inhibited the migration of cervical cancer cells (Fig. 8I, J). Transwell assay showed that knockdown of *SFT2D1 *significantly inhibited the migratory and invasive abilities of SiHa and CaSki cells (Fig. 8K, L).

## Discussion

In this study, we developed and validated a prognostic model based on CuRA genes in cervical cancer. We also analyzed clinical characteristics and the immune microenvironment between the high and low-CuRA patient groups. Based on the modeling scores, we analyzed drug sensitivity of the high or low-CuRA patient group to provide guidance on drug administration. *SFT2D1*, as a key gene involved in the progression of cervical cancer, it was associated with the cuproptosis-dependent angiogenesis pathway.

Cuproptosis and tumor angiogenesis are closely linked in the tumor microenvironment. CuRA genes may help explain the potential link between cuproptosis and angiogenesis, which could improve the prognosis of cervical cancer patients. We constructed and validated our model based on CuRA signatures using patient data from TCGA and GEO. Several web tools enable us to extract prognostic variable characteristics from multi-omics data by selecting clinical variables or subgroup variables (lasso, elastic network regularization, and network regularized high-dimensional Cox regression) [44]. This implies the necessity of choosing the optimal regression method for subsequent studies. After comparing the errors of these three regression methods, we observed that lasso regression minimized the error. Consequently, we have opted for the lasso regression method to construct the CuRA model. The AUC values for 1, 2, 3, and 5-year survival in TCGA patients were 0.653, 0.759, 0.748, and 0.799, respectively, while the AUC values for 2, 3, and 5-year survival in GEO patients were 0.653, 0.646, and 0.604, respectively. The shorter survival time of patients in the high CuRA group may indicate that tumor cells promote tumor progression through cuproptosis-associated angiogenesis. In the analysis of clinical characteristics, CuRA modeling scores, N_stage, and T_stage were independent risk factors, suggesting that modeling scores could independently contribute to cervical cancer progression as a risk factor. Our CuRA model is represented by 10 genes involved in programmed death-related pathways, membrane vesicle transport, and tumorigenesis and progression. Among them, SFT2 Domain Containing 1 (SFT2D1) is involved in protein and vesicle-mediated translocation and is also associated with poor survival in patients with high-risk neuroblastoma [45]. GSEA pathway analysis results suggest that *SFT2D1 *plays an important role in tumor-related pathways and is associated with the invasion and progression of cervical cancer. The immune microenvironment was scored for *SFT2D1*, and patients with high *SFT2D1 *expression had a higher CuRA score and a worse prognosis, showing a more aggressive immunosuppressive phenotype. We verified *SFT2D1 *was significantly upregulated in cervical cancer cells by western blotting, RT-qPCR, and immunohistochemistry. Therefore, *SFT2D1*, a CuRA modeling gene, may serve as a marker gene and provide a new reference for the treatment of cervical cancer patients.

Antitumor strategies targeting angiogenesis have been used in the clinical management of patients with metastatic or recurrent cervical cancer. However, improvements in overall survival (OS) and progression-free survival (PFS) times for patients are still limited. Combining immune checkpoint inhibitors (ICIs) with vascular targeting therapy has demonstrated synergistic sensitization in the treatment of various tumors, including hepatocellular carcinoma [46, 47], Non-small cell lung cancer [48], gastric cancer [49]. In cervical cancer, a phase III randomized controlled trial showed increased overall survival in patients with recurrent or metastatic cervical cancer after treatment with pablizumab combined with chemotherapy and bevacizumab [50]. Therefore, researching the differences in immune microenvironment in cervical cancer patients with different CuRA scores can help promote the precise combination of immunotherapy and vascular targeting therapy, and enable personalized treatment selection of immune checkpoint inhibitors for patients with CuRA-related cervical cancer. Results showed that the group of patients with high-CuRA scores exhibited immunosuppression, while patients with low-CuRA scores may have a more significant therapeutic effect with immune agents targeting CD8 + T cells compared to patients in the high-CuRA scores group. Patients with high-CuRA scores had lower expression in most immune checkpoint genes. However, a minority of immune checkpoint genes presented high expression in the high-CuRA group, which suggests that treatment with anti-*IL10RB*, anti-*KDR*, anti*-TGFB1*, and anti-*TGFBR1* may be considered to improve prognosis of patients in the high-CuRA group.

Cuproptosis plays an important role in tumor cell proliferation and angiogenesis. Cell-cell communication analysis based on single-cell sequencing helps to reveal the tumor immune microenvironment and changes in the tumor itself [51]. Previous studies have shown that *FDX1*, as a key gene in cuproptosis, is involved in the progression of hepatocellular carcinoma [52] and glioma [53] as well as tumor immunity and drug sensitivity [54]. To investigate the relationship between angiogenesis and cuproptosis in cervical cancer, we identified two types of cells, FDX1 + tumor/epithelial cells and VEGFA + tumor/epithelial cells, and conducted cell-cell communication analysis. We found that the genes *VEGFA *and *PGF*, which promote angiogenesis, are highly expressed in these two types of cells. Meanwhile, both types of cells are sent as signals to endothelial cells, indicating that they can affect the process of transendothelial migration and promote angiogenesis. In addition, both types of cells can transmit cell signals to macrophages to affect the macrophage migration inhibitory factor (MIF) pathway. Previous studies have shown that MIF is involved in multiple immune processes and mediates immune escape leading to tumor metastasis [55–58]. Our results suggest that VEGFA + tumor/epithelial cells and FDX1 + tumor/epithelial cells may play irreplaceable roles in the tumor immune microenvironment.

We conducted drug sensitivity analysis for different CuRA groups of patients, which may lay the foundation for personalized treatment. The GDSC provides information on the sensitivity of tumor cell lines to drugs, with a smaller IC50 indicating greater sensitivity of the biological system to the compound. Our analysis showed that among small molecule tyrosine kinase inhibitors, imatinib, pazopanib, and sorafenib may provide better efficacy for patients with high CuRA scores, while AMG.706, CEP.701, and sunitinib may provide better efficacy for patients with lower CuRA scores. Receptor tyrosine kinase inhibitors have significant anti-angiogenic effects and have made great progress in the treatment of gynecological tumors [59]. For example, pazopanib has improved PFS and OS in patients with advanced or recurrent cervical cancer, while AMG.706 (motesanib) inhibits angiogenesis in recurrent ovarian cancer and CEP.701 (lestaurtinib) inhibits the growth of cervical cancer cells [60–62]. Studies have shown differences in IC50 values of drugs such as pazopanib, sorafenib, sunitinib, and imatinib among different CuRA subgroups of clear cell renal cell carcinoma, bladder cancer, and triple-negative breast cancer [63–65]. Our study also suggested that in the high CuRA score group, IC50 values of PARP inhibitors ABT.888 (Veliparib), AZD.2281 (Olaparib), and MS.275 (Entinostat) were higher, indicating that these drugs were effective bythe cuproptosis-dependent angiogenesis pathway These studies supported our results of drug sensitivity analysis and suggested that research on anti-cuproptosis-related angiogenesis targeted drugs may bring new treatment ideas and expand the application of drugs in the current dilemma.

In conclusion, we have conducted a novel cuproptosis-related angiogenesis (CuRA) gene signature using single-cell RNA sequencing and bulk RNA sequencing data, which provides significant predictive value for patients with cervical cancer. Of course, there are limitations to our results, and future relevant mechanisms need to be further validated in in vivo and in vitro experiments.

### Electronic supplementary material

Below is the link to the electronic supplementary material.

**Supplementary Material 1: Figure S1:** Forest plot of 66 CuRA prognostic genes by univariate COX analysis. **Figure S2:** Comparison of errors in ridge regression, lasso regression and elastic network regression and comprehensive comparison of the errors in three regression algorithms. **Figure S3:** Construction and verification of a CuRA prognostic model. **Figure S4:** Localization and validation of 10 modeling genes in the GSE168652 dataset. **Figure S5:** Immune infiltration landscape in high and low CuRA-groups calculated by CIBERSORT, EPIC, MCP, Quanti-seq, TIMER, xCell algorithms. **Figure S6:** Differences in mutation frequency between high and low CuRA groups. **Figure S7:** Survival analysis of 10 modeling genes. **Figure S8:** Assessment of regulatory pathways and immune microenvironment of *SFT2D1*. **Figure S9:** Visual circular and hierarchical plots showing cellular communication in the VEGF pathway. **Figure S10:** Cell Communication Analysis

**Supplementary Material 2: Table S1:** Genelist of Cuproptosis-Related Gene. **Table S2:** Genelist of Cuproptosis-Related Angiogenesis Gene (CuRA). **Table S3:** Univariate Analysis and Multivariate Analysis of SFT2D1 and Clinical Characteristics. **Table S4:** Prediction of small molecule drugs by modeling genes. **Table S5: **GSEA to assess SFT2D1-related pathways. **Table S6:** Oligonucleotides used in research

## Data Availability

The data and materials in the current study are available under the permission of author.
