# Supplementary material for: Identification of a new gene signature for prognostic evaluation in cervical cancer: based on cuproptosis-associated angiogenesis and multi-omics analysis
Source: Cancer Cell Int. 2024 Jan 10;24:23. doi: 10.1186/s12935-023-03189-x (PMC10782580; doi:10.1186/s12935-023-03189-x)
Supplement: Supplementary file 1 — Supplementary Material 1: Figure S1: Forest plot of 66 CuRA prognostic genes by univariate COX analysis. Figure S2: Comparison of errors in ridge regression, lasso regression and elastic network regression and comprehensive comparison of the errors in three regression algorithms. Figure S3: Construction and verification of a CuRA prognostic model. Figure S4: Localization and validation of 10 modeling genes in the GSE168652 dataset. Figure S5: Immune infiltration landscape in high and low CuRA-groups calculated by CIBERSORT, EPIC, MCP, Quanti-seq, TIMER, xCell algorithms. Figure S6: Differences in mutation frequency between high and low CuRA groups. Figure S7: Survival analysis of 10 modeling genes. Figure S8: Assessment of regulatory pathways and immune microenvironment of SFT2D1. Figure S9: Visual circular and hierarchical plots showing cellular communication in the VEGF pathway. Figure S10: Cell Communication Analysis [file 12935_2023_3189_MOESM1_ESM.docx]

**Supplementary Figures**


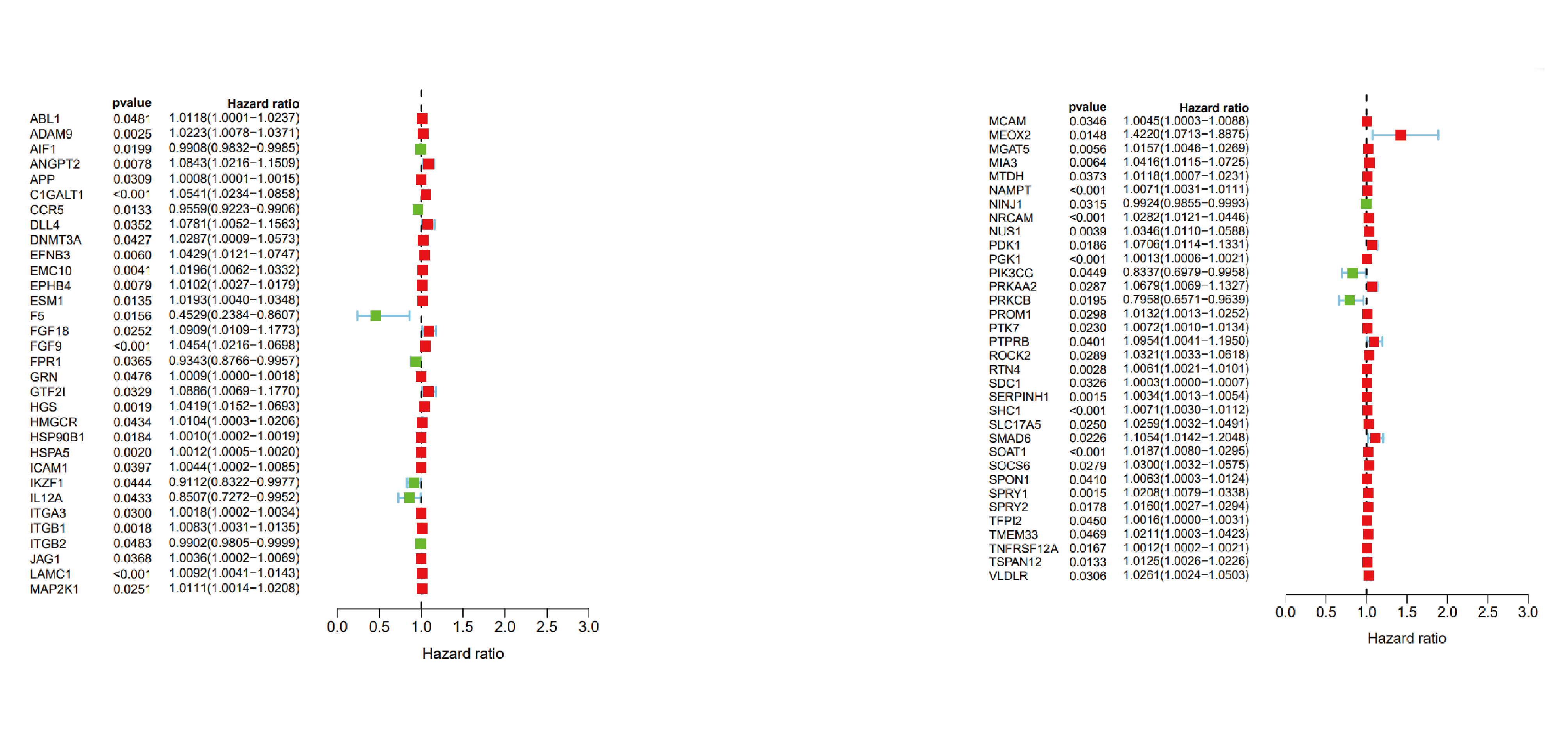


**Figure S1** Forest plot of 66 CuRA prognostic genes by univariate COX analysis.


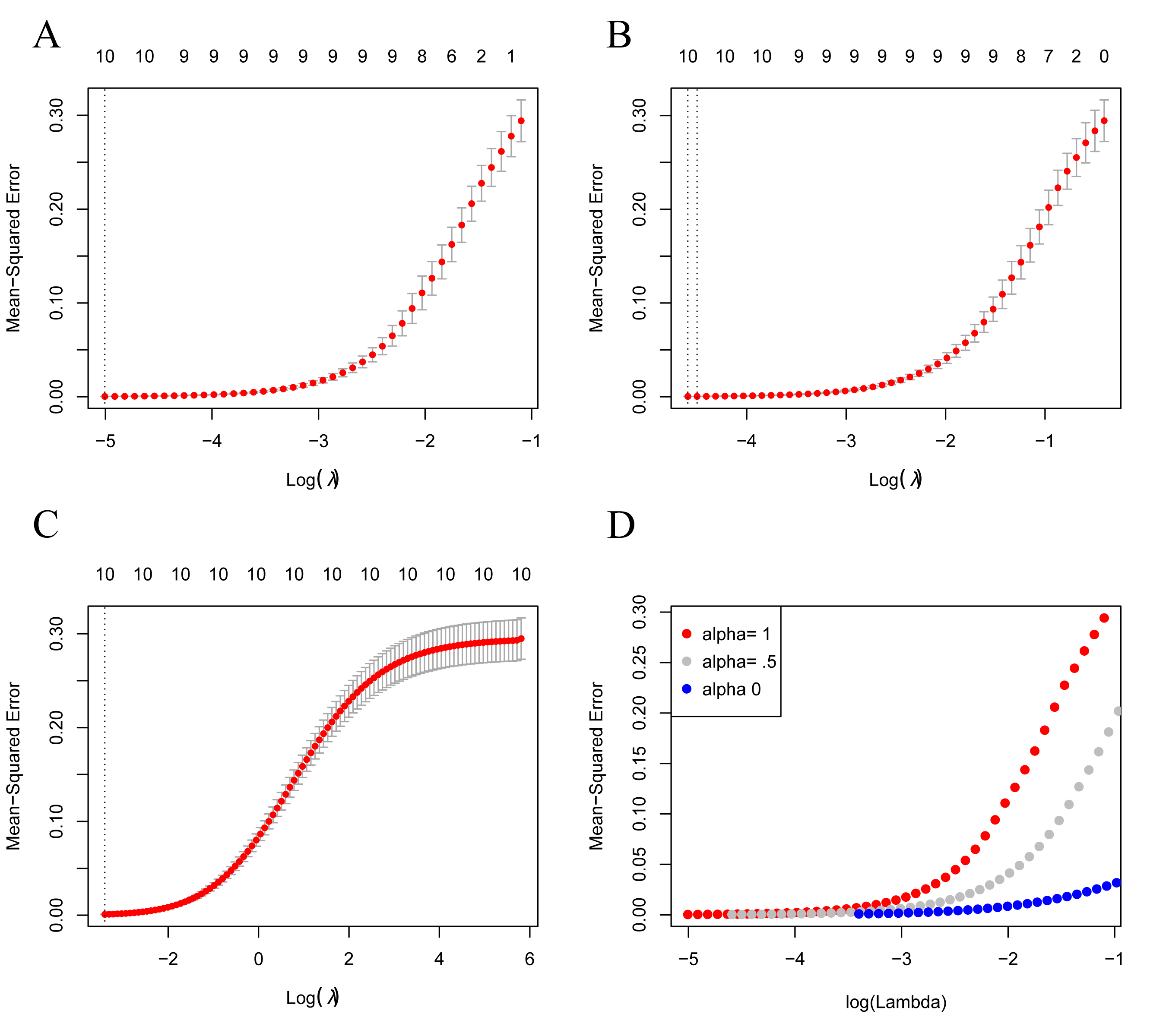
**Figure S2** Comparison of errors in ridge regression (A), lasso regression (B) and elastic network regression (C) and comprehensive comparison of the errors in three regression algorithms (D).


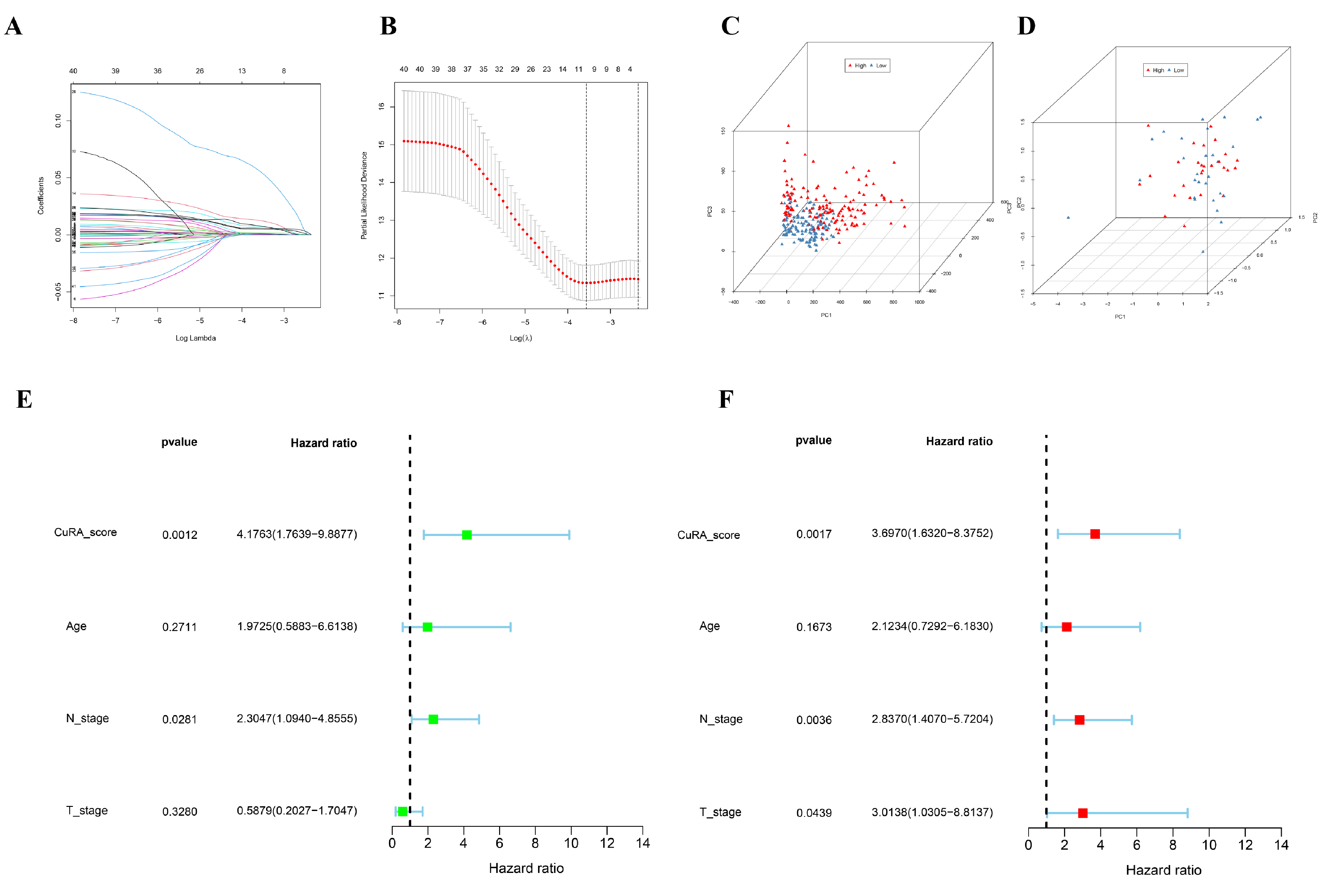


**Figure S3** Construction and verification of a CuRA prognostic model. (A-B) Modeling of optimal gene number selection. PCA distribution analysis of TCGA(C) and GEO(D) samples. (E) Clinical characteristics analysis by univariate Cox analysis. (F) Clinical characteristics analysis by multivariate Cox analysis.


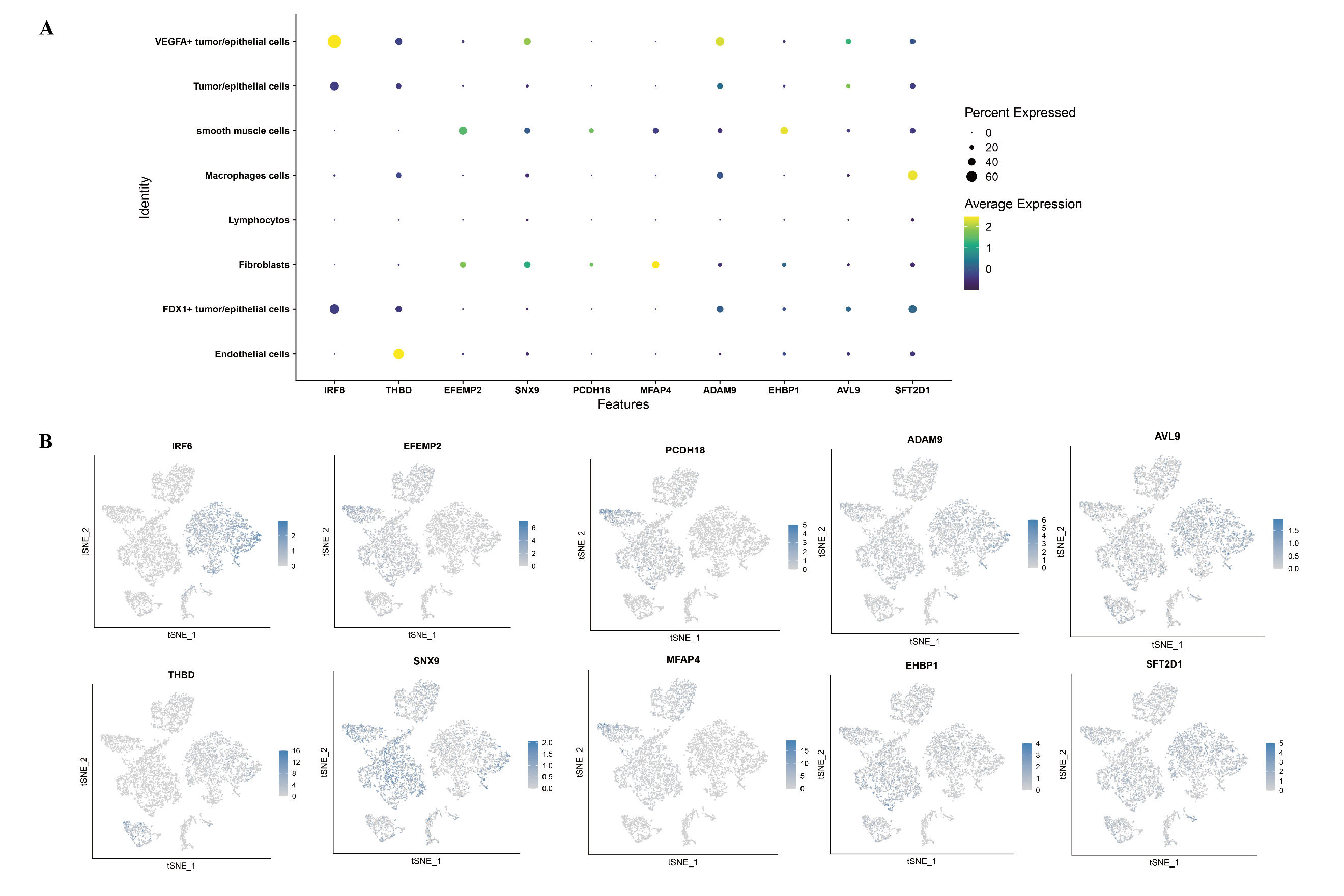


**Figure S4** Localization and validation of 10 modeling genes in the GSE168652 dataset. (A-B) IRF6 was mainly expressed in VEGF+ epithelial/tumor cells. THBD was mainly expressed in endothelial cells. EFEMP2 was mainly expressed in smooth muscle cells, fibroblasts. SNX9, PCDH18, MFAP4 were mainly expressed in fibroblasts, smooth muscle cells. ADAM9 was mainly expressed in VEGF+ epithelial/tumor cells, FDX1+ epithelial/tumor cells. EHBP1 was mainly expressed in smooth muscle cells. AVL9 was mainly expressed in VEGF+ epithelial/tumor cells and other epithelial/tumor cells. SFT2D1 was mainly expressed in macrophages, FDX1+ epithelial/tumor cells.

**
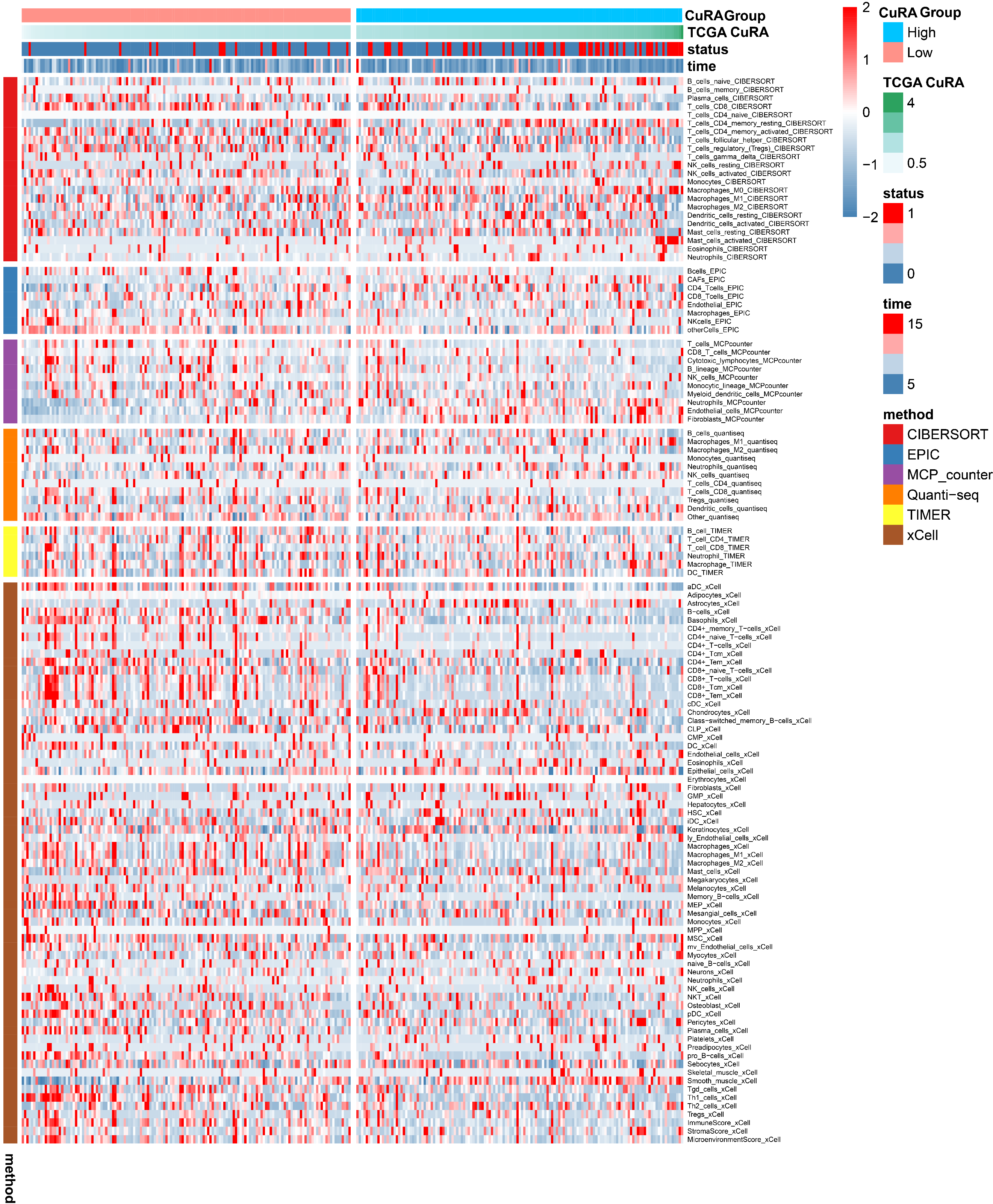
**

**Figure S5** Immune infiltration landscape in high and low CuRA-groups calculated by CIBERSORT, EPIC, MCP, Quanti-seq, TIMER, xCell algorithms.


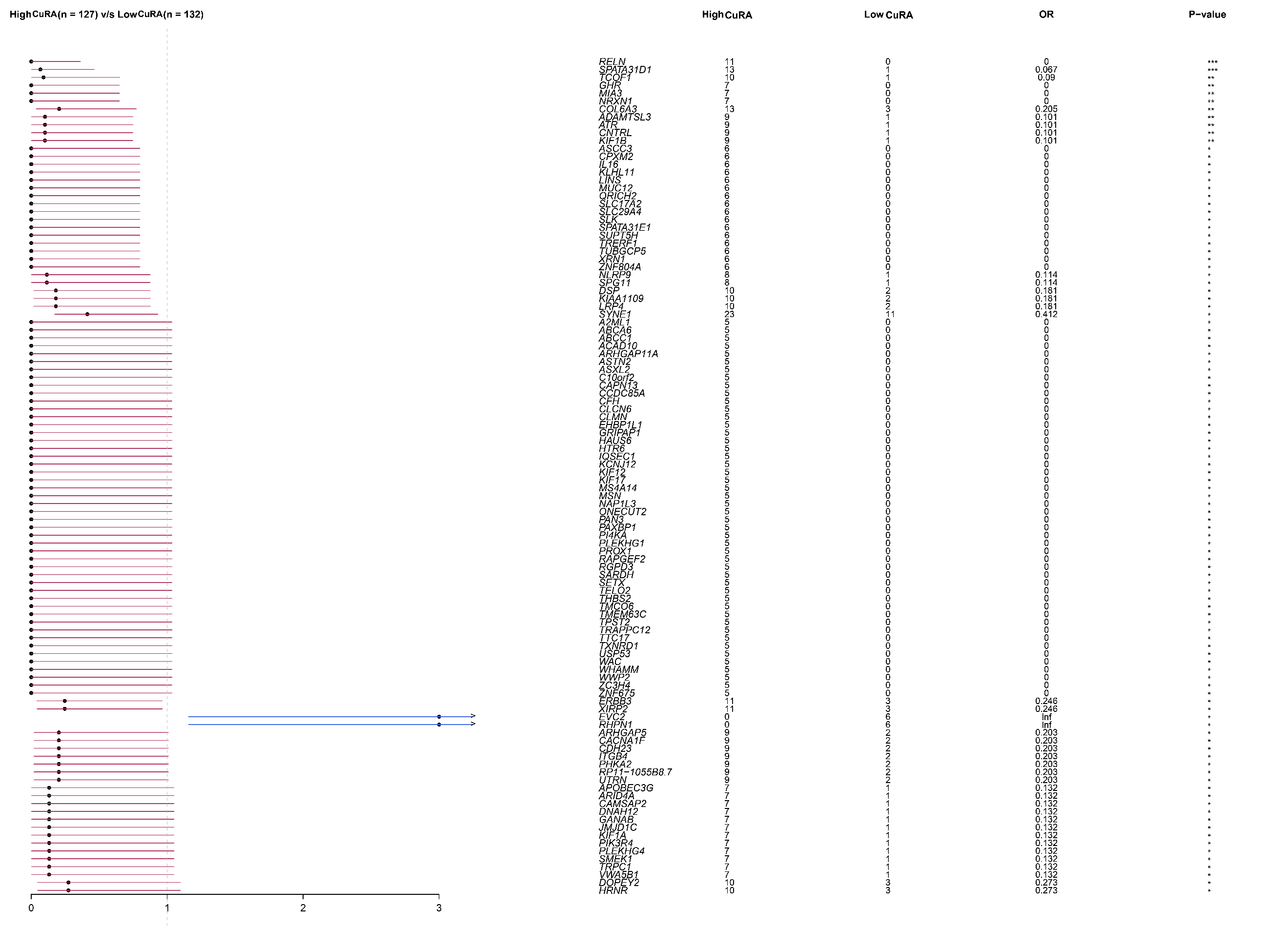


**Figure S6** Differences in mutation frequency between high and low CuRA groups. Odds ratio with 95% CI. (1=no effect, <1 High CuRA has more mutants). * *P* < 0.05, ** *P* < 0.01, *** *P* < 0.001.


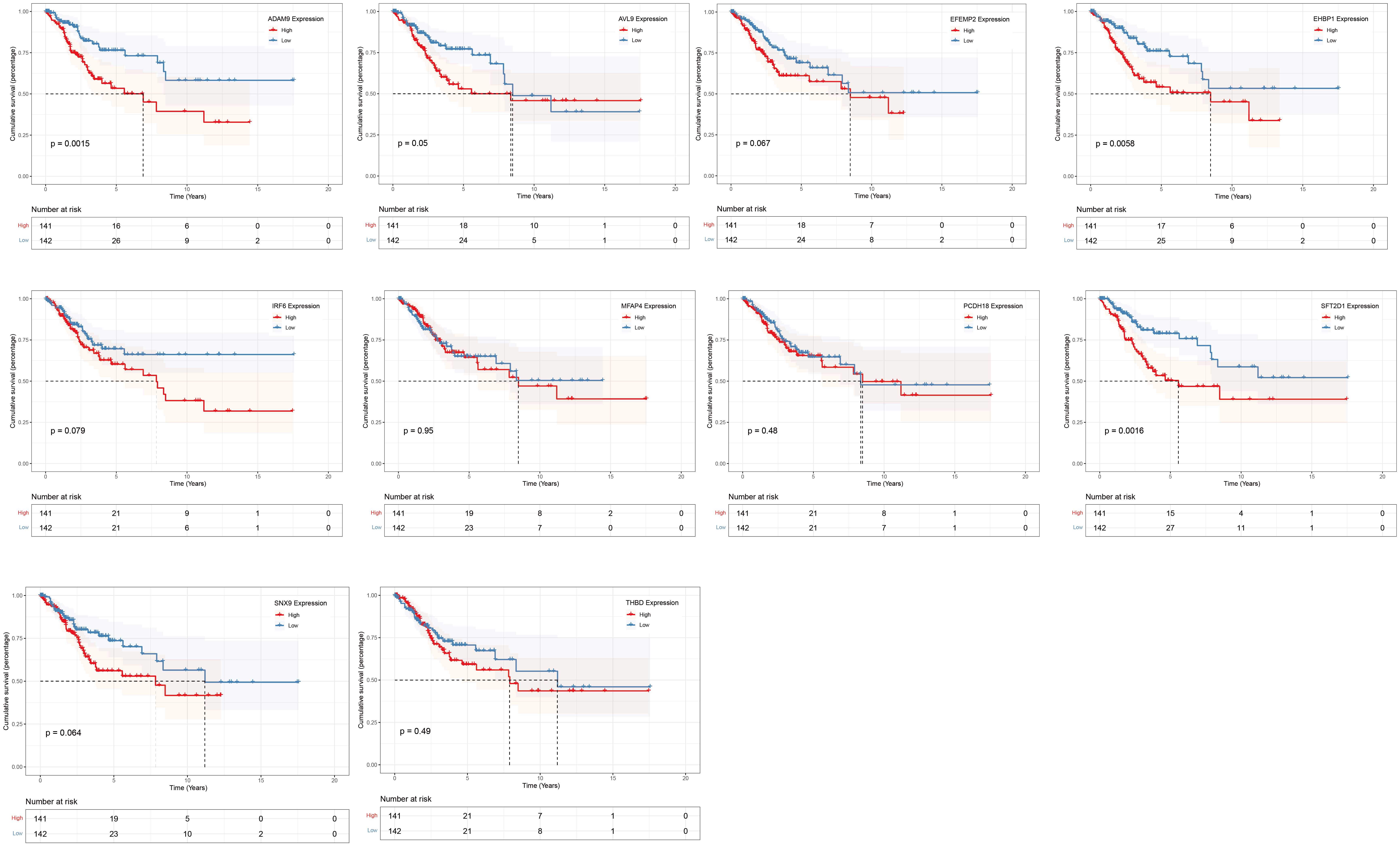


**Figure S7** Survival analysis of 10 modeling genes.


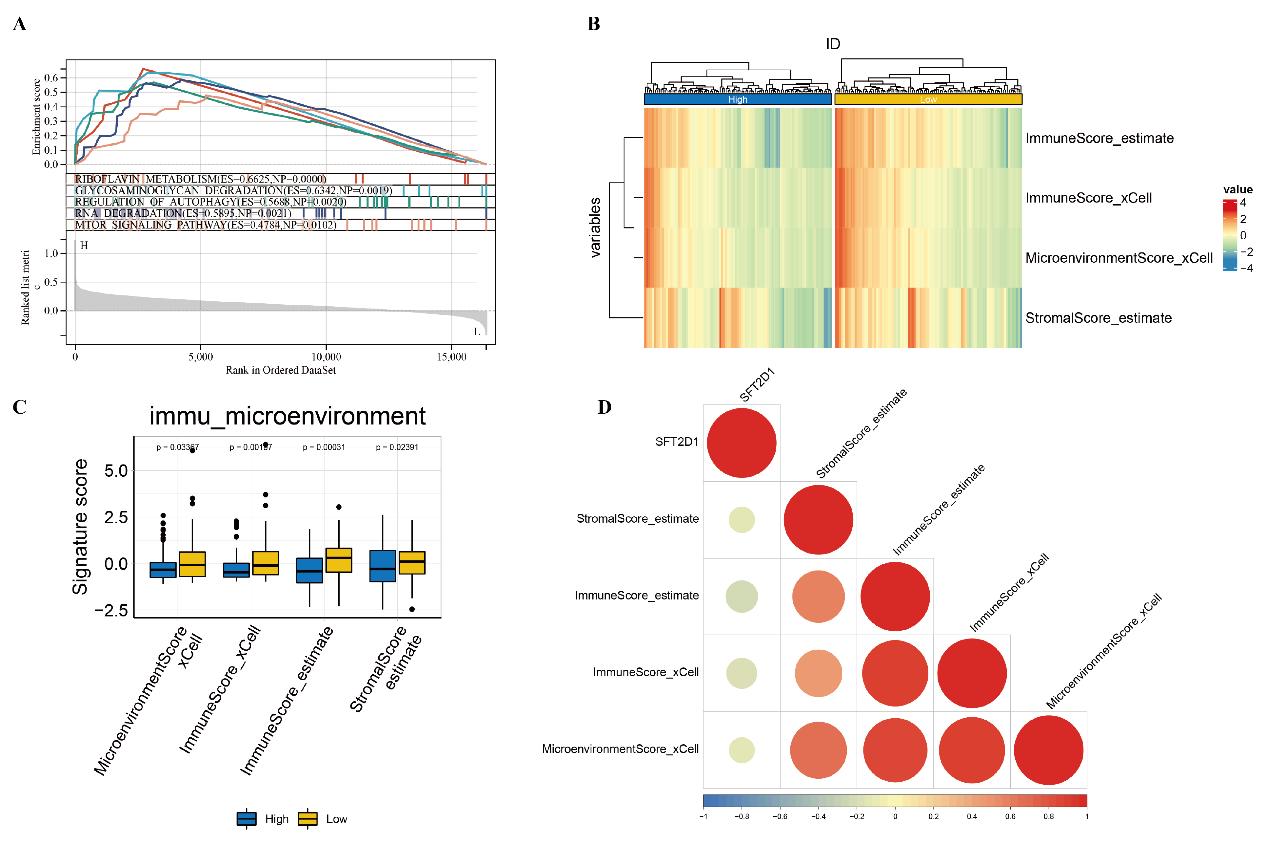


**Figure S8** Assessment of regulatory pathways and immune microenvironment of *SFT2D1*. (A) GSEA analysis of *SFT2D1*, showing the significant 5 pathways. (B) Histogram of immune microenvironment scores of *SFT2D1*. (C) Heatmap of immune microenvironment scores of *SFT2D1*. (D) Correlation between *SFT2D1* expression and immune microenvironment scores.


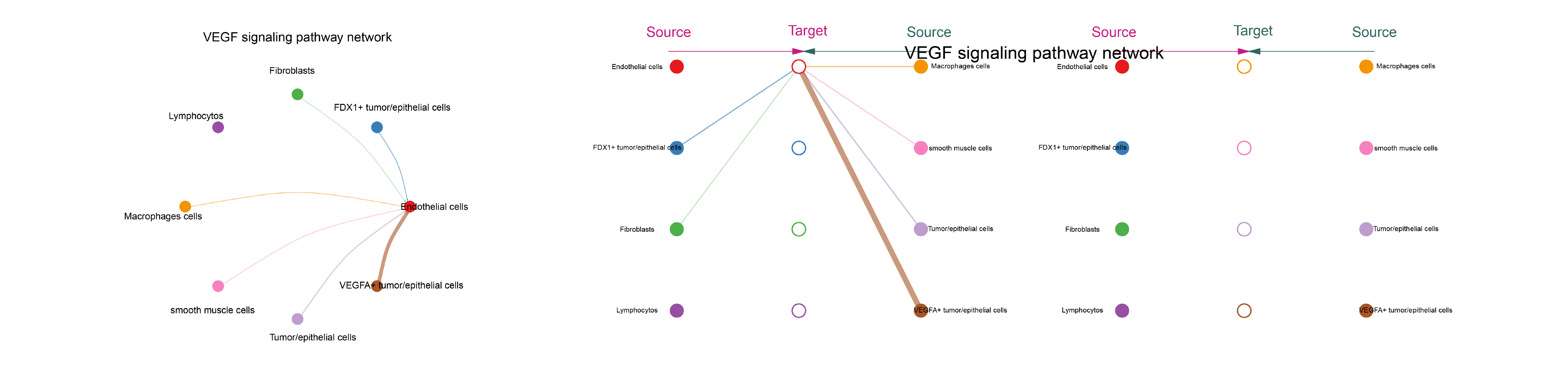


**Figure S9** Visual circular and hierarchical plots showing cellular communication in the VEGF pathway.

**
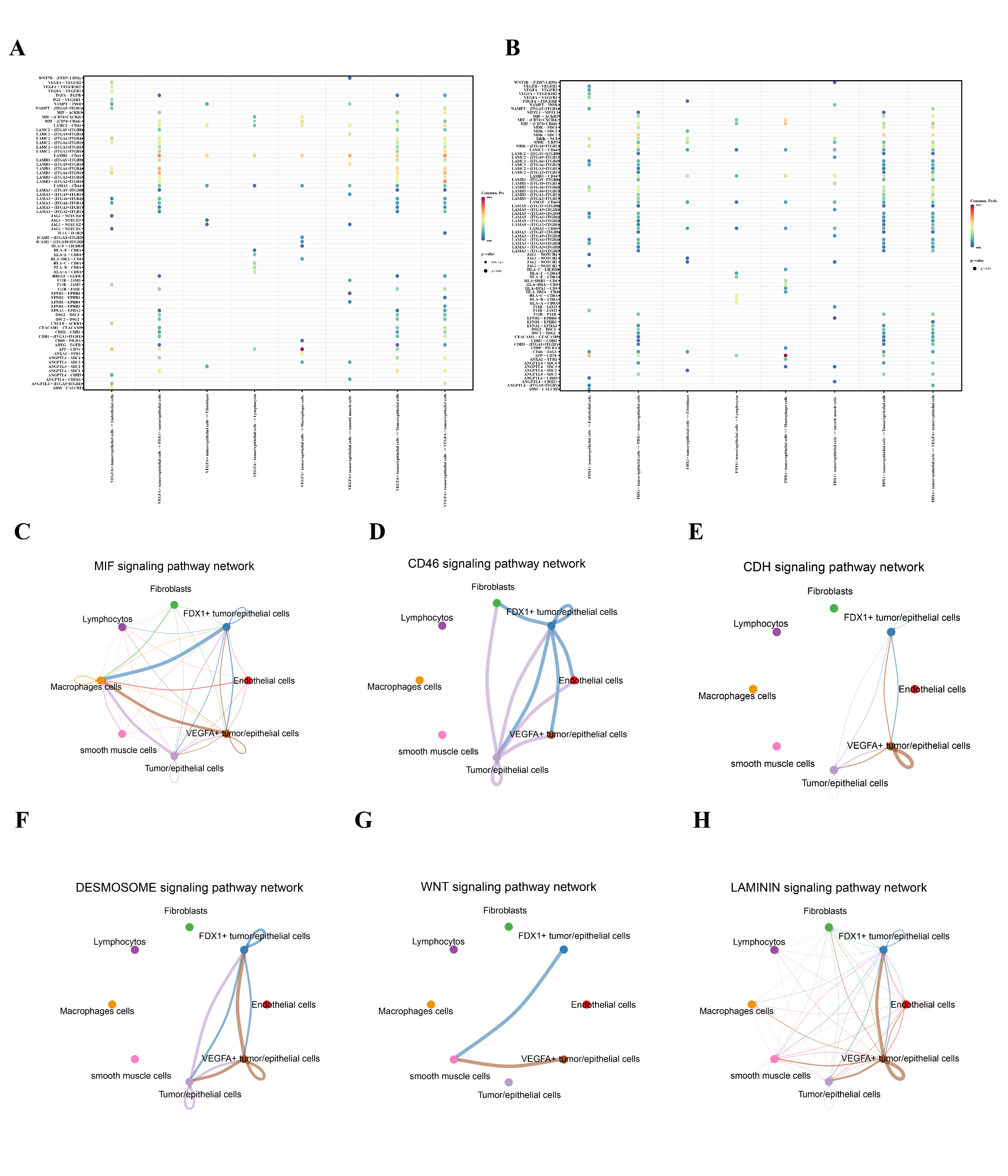
**

**Figure S10** Cell Communication Analysis. (A) Analysis of relevant receptor-ligand pairs for communication between VEGF+ tumor/epithelial cells and eight types of cells. (B) Analysis of relevant receptor-ligand pairs for communication between FDX1+ tumor/epithelial cells and eight types of cells. (C-H) Intercellular communication of 8 cell types in MIF, CD46, CDH, DESMOSOME, WNT, LAMININ signaling pathways, respectively.
