# Supplementary material for: Identification of a new gene signature for prognostic evaluation in cervical cancer: based on cuproptosis-associated angiogenesis and multi-omics analysis
Source: Cancer Cell Int. 2024 Jan 10;24:23. doi: 10.1186/s12935-023-03189-x (PMC10782580; doi:10.1186/s12935-023-03189-x)
Supplement: Supplementary file 2 — Supplementary Material 2: Table S1: Genelist of Cuproptosis-Related Gene. Table S2: Genelist of Cuproptosis-Related Angiogenesis Gene (CuRA). Table S3: Univariate Analysis and Multivariate Analysis of SFT2D1 and Clinical Characteristics. Table S4: Prediction of small molecule drugs by modeling genes. Table S5: GSEA to assess SFT2D1-related pathways. Table S6: Oligonucleotides used in research [file 12935_2023_3189_MOESM2_ESM.docx]

**Supplementary Tables**

**Table S1 Genelist of Cuproptosis-Related Gene**

| Gene name |
| --- |
| NFE2L2  NLRP3  ATP7B  ATP7A  SLC31A1  FDX1  LIAS  LIPT1  LIPT2  DLD  DLAT  PDHA1  PDHB  MTF1  GLS  CDKN2A  DBT  GCSH  DLST |

**Table S2 Genelist of Cuproptosis-Related Angiogenesis Gene（CuRA）**

| Gene Symbol |
| --- |
| ABL1 |
| DLL4 |
| EFNB3 |
| ITGA3 |
| TNFRSF12A |
| GRN |
| MCAM |
| PGK1 |
| MEOX2 |
| TMEM33 |
| HMGCR |
| SDC1 |
| PTPRB |
| ROCK2 |
| LAMC1 |
| SPRY2 |
| ITGB2 |
| SPRY1 |
| ESM1 |
| SPON1 |

**Table S3 Univariate Analysis and Multivariate Analysis of SFT2D1 and Clinical Characteristics**

| Characteristics | Total(N) | Univariate analysis | |  | Multivariate analysis | |
| --- | --- | --- | --- | --- | --- | --- |
|  |  | Hazard ratio (95% CI) | P value |  | Hazard ratio (95% CI) | P value |
| Pathologic T stage | 243 |  | **< 0.001** |  |  |  |
| T1 | 140 | Reference |  |  | Reference |  |
| T2 | 72 | 1.140 (0.557 - 2.333) | 0.720 |  | 0.408 (0.138 - 1.204) | 0.105 |
| T3&T4 | 31 | 4.019 (2.072 - 7.797) | **< 0.001** |  | 3.940 (1.484 - 10.456) | **0.006** |
| Pathologic N stage | 195 |  | **0.003** |  |  |  |
| N0 | 134 | Reference |  |  | Reference |  |
| N1 | 61 | 2.844 (1.446 - 5.593) | **0.002** |  | 3.979 (1.890 - 8.376) | **< 0.001** |
| SFT2D1 | 306 |  | **0.011** |  |  |  |
| Low | 153 | Reference |  |  | Reference |  |
| High | 153 | 1.844 (1.141 - 2.982) | **0.012** |  | 2.123 (1.014 - 4.445) | **0.046** |

**Table S4 Prediction of small molecule drugs by modeling genes**

| Term | P-value | Odds Ratio | Combined Score | Genes |
| --- | --- | --- | --- | --- |
| maltotetraose BOSS | 4.70E-05 | 262.7763 | 2618.598 | EFEMP2; IRF6 |
| MINOCYCLINE HYDROCHLORIDE BOSS | 4.46E-04 | 80.35484 | 619.9225 | EFEMP2; IRF6 |
| progesterone CTD 00006624 | 1.15E-04 | 14.19895 | 128.8372 | THBD; MFAP4; ADAM9; IRF6; PCDH18; EHBP1 |
| tolnaftate PC3 DOWN | 0.006056 | 20.7479 | 105.953 | IRF6; AVL9 |
| Retinoic acid CTD 00006918 | 0.001283 | 8.636406 | 57.50575 | THBD; MFAP4; EFEMP2; ADAM9; IRF6; PCDH18; EHBP1 |
| VALPROIC ACID CTD 00006977 | 0.015985 | 5.627935 | 23.27758 | MFAP4; EFEMP2; ADAM9; SNX9; IRF6; AVL9; PCDH18; EHBP1 |

**Table S5 GSEA to assess SFT2D1-related pathways**

| Term | ES | NES | pvalue | FDR | FWER |
| --- | --- | --- | --- | --- | --- |
| N_GLYCAN_BIOSYNTHESIS | 0.643 | 1.9418 | 0 | 0.0988 | 0.07 |
| RIBOFLAVIN_METABOLISM | 0.6625 | 1.9642 | 0.002 | 0.1481 | 0.051 |
| PURINE_METABOLISM | 0.4028 | 1.6557 | 0.002 | 0.2252 | 0.606 |
| RNA_DEGRADATION | 0.5895 | 1.8322 | 0.0021 | 0.1172 | 0.228 |
| REGULATION_OF_AUTOPHAGY | 0.5688 | 1.8884 | 0.0041 | 0.0976 | 0.134 |
| PATHOGENIC_ESCHERICHIA_COLI_INFECTION | 0.6003 | 1.9146 | 0.0042 | 0.0952 | 0.1 |
| UBIQUITIN_MEDIATED_PROTEOLYSIS | 0.4832 | 1.6892 | 0.006 | 0.2397 | 0.541 |
| CYSTEINE_AND_METHIONINE_METABOLISM | 0.5166 | 1.7043 | 0.0081 | 0.2856 | 0.498 |
| THYROID_CANCER | 0.5399 | 1.6909 | 0.0082 | 0.2592 | 0.538 |
| GLYCOSPHINGOLIPID_BIOSYNTHESIS_GANGLIO_SERIES | 0.7789 | 1.6687 | 0.0096 | 0.2377 | 0.575 |
| MTOR_SIGNALING_PATHWAY | 0.4784 | 1.6495 | 0.0107 | 0.2082 | 0.625 |
| GLYCOSAMINOGLYCAN_DEGRADATION | 0.6342 | 1.8527 | 0.0118 | 0.1142 | 0.189 |
| VIBRIO_CHOLERAE_INFECTION | 0.4463 | 1.6022 | 0.0123 | 0.2209 | 0.707 |
| OTHER_GLYCAN_DEGRADATION | 0.6612 | 1.6969 | 0.0128 | 0.2708 | 0.516 |
| LYSOSOME | 0.4582 | 1.6545 | 0.0185 | 0.2132 | 0.61 |
| PROGESTERONE_MEDIATED_OOCYTE_MATURATION | 0.436 | 1.6124 | 0.0199 | 0.2265 | 0.689 |
| SNARE_INTERACTIONS_IN_VESICULAR_TRANSPORT | 0.7088 | 1.6638 | 0.0202 | 0.2293 | 0.594 |
| PROTEIN_EXPORT | 0.675 | 1.6483 | 0.0235 | 0.1981 | 0.628 |
| NUCLEOTIDE_EXCISION_REPAIR | 0.5446 | 1.6701 | 0.0261 | 0.2546 | 0.573 |
| SPLICEOSOME | 0.5674 | 1.7254 | 0.0278 | 0.2737 | 0.455 |
| OOCYTE_MEIOSIS | 0.4234 | 1.5275 | 0.0348 | 0.2756 | 0.838 |
| NOTCH_SIGNALING_PATHWAY | 0.4246 | 1.5471 | 0.038 | 0.2835 | 0.811 |
| LYSINE_DEGRADATION | 0.4736 | 1.516 | 0.0387 | 0.2764 | 0.85 |
| AMINOACYL_TRNA_BIOSYNTHESIS | 0.5962 | 1.6214 | 0.0413 | 0.2254 | 0.675 |
| COLORECTAL_CANCER | 0.4361 | 1.5113 | 0.0418 | 0.2674 | 0.858 |
| HOMOLOGOUS_RECOMBINATION | 0.576 | 1.6053 | 0.0432 | 0.2265 | 0.7 |
| BIOSYNTHESIS_OF_UNSATURATED_FATTY_ACIDS | 0.5275 | 1.4901 | 0.0447 | 0.278 | 0.892 |
| GLYCOSAMINOGLYCAN_BIOSYNTHESIS_KERATAN_SULFATE | 0.6107 | 1.5329 | 0.0448 | 0.2763 | 0.833 |
| BASE_EXCISION_REPAIR | 0.5337 | 1.5785 | 0.0451 | 0.2486 | 0.756 |
| PROPANOATE_METABOLISM | 0.4878 | 1.5135 | 0.0488 | 0.2721 | 0.855 |
| SELENOAMINO_ACID_METABOLISM | 0.5389 | 1.5553 | 0.0492 | 0.2795 | 0.798 |

**Table S6 Oligonucleotides used in research**

| **Oligonucleotides** | **Nucleotide sequence (5'-3')** |
| --- | --- |
| **SiRNA** |  |
| siRNA (SFT2D1-homo-341) | GCUCUUUGGUGGCAUAAGATT |
| siRNA (SFT2D1-homo-72) | CCUCAUCCCUUAGUUUCAATT |
| siRNA (SFT2D1-homo-424) | CAUCCCAUAUGCAAGGGAUTT |
| siRNA (SFT2D1-homo-281) | GCAACAAGAUUGCUUGCAATT |
|  |  |
| **Primer** |  |
| GAPDH | CAGGAGGCATTGCTGATGAT (forward) |
|  | GAAGGCTGGGGCTCATTT (reverse) |
| SFT2D1 | TGCCTCATCCCTTAGTTTCAACACC (forward) |
|  | AGCCACAGCAATCCAGTTCCAAG (reverse) |
